# Lithographic Processes for the Scalable Fabrication of Micro- and Nanostructures for Biochips and Biosensors

**DOI:** 10.1021/acssensors.0c02704

**Published:** 2021-04-08

**Authors:** Silvia Fruncillo, Xiaodi Su, Hong Liu, Lu Shin Wong

**Affiliations:** † Manchester Institute of Biotechnology, University of Manchester, 131 Princess Street, Manchester, M1 7DN, United Kingdom; ‡ Department of Chemistry, University of Manchester, Oxford Road, Manchester M13 9PL, United Kingdom; § Institute of Materials Research and Engineering, Agency for Science, Technology and Research (A*STAR), 2 Fusionopolis Way, #08-03, Innovis, Singapore 138634, Singapore; ∥ Department of Chemistry, National University of Singapore, Block S8, Level 3, 3 Science Drive, Singapore 117543, Singapore

**Keywords:** large-scale lithography, biosensors, biochips, high throughput, DNA microarray, protein array, high resolution, plasmonic, nanopore sensors, electrochemical sensing

## Abstract

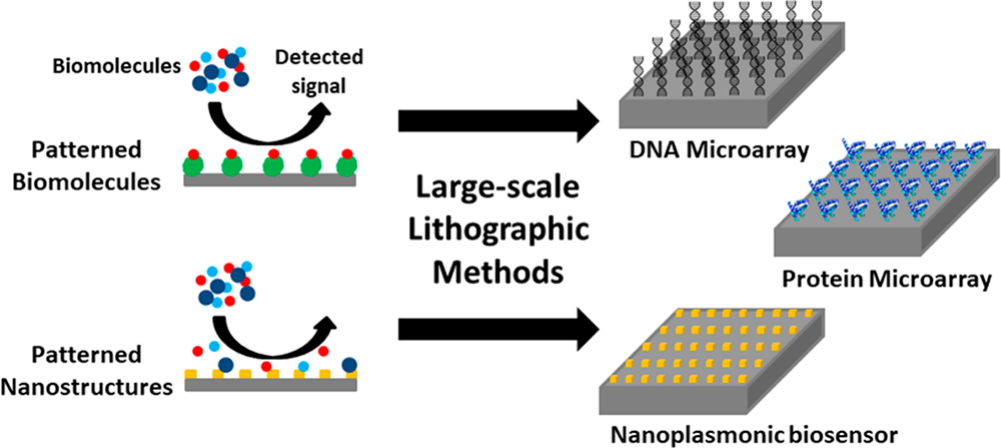

Since the early 2000s, extensive research has been performed to address numerous challenges in biochip and biosensor fabrication in order to use them for various biomedical applications. These biochips and biosensor devices either integrate biological elements (e.g., DNA, proteins or cells) in the fabrication processes or experience post fabrication of biofunctionalization for different downstream applications, including sensing, diagnostics, drug screening, and therapy. Scalable lithographic techniques that are well established in the semiconductor industry are now being harnessed for large-scale production of such devices, with additional development to meet the demand of precise deposition of various biological elements on device substrates with retained biological activities and precisely specified topography. In this review, the lithographic methods that are capable of large-scale and mass fabrication of biochips and biosensors will be discussed. In particular, those allowing patterning of large areas from 10 cm^2^ to m^2^, maintaining cost effectiveness, high throughput (>100 cm^2^ h^–1^), high resolution (from micrometer down to nanometer scale), accuracy, and reproducibility. This review will compare various fabrication technologies and comment on their resolution limit and throughput, and how they can be related to the device performance, including sensitivity, detection limit, reproducibility, and robustness.

In the past 20 years, increasing attention has been turned to the fabrication of “biodevices” such as biological arrays and biosensor devices, where biomolecules are integrated with conventional inorganic components consisting of metallic, semiconductor, and dielectric materials.^1^ In such devices, an external stimulus (e.g., biomolecule binding, pH change, etc.) leads to a change in the physicochemical properties at the biomolecule–device interface, which in turn transduces a signal that can be detected and quantified spectroscopically or electronically.^2−6^ Here, biological arrays can be generally defined as surface substrates upon which are placed arrays of micrometer-scale features consisting of biological components (e.g., DNA, protein, or cells). These arrays are sometimes colloquially referred to as “biochips” and are widely employed for the parallelized (and thus high-throughput) detection of biomolecular and cellular interactions. They have thus found applications in basic research for cell and molecular biology, clinical diagnostics (genotyping and biomarker identification), drug screening, and tissue engineering.^7−14^ Biosensors instead are devices that include a biological component in the fabrication process but are used for the sensing of biomolecules or biological phenomena.^15−17^ For example, biosensors that generate an electronic output (“bioelectronic” devices) are also widely used in molecular sensing for health monitoring. For example, amperometric blood glucose meters based on immobilized glucose oxidase (GOx) have long been commercialized for diabetic self-monitoring.^18^

A key aspect in the development of biochips and biosensor devices for practical applications is the ability to manufacture them on a large scale and at acceptable cost and time scales. Indeed, scalable manufacturing with high reproducibility is required to produce chips or devices for large-scale deployment and validation (e.g., for clinical use^8^). Moreover, another important aspect of device development is the capability to generate micro- and nanostructures, to provide desirable properties such as plasmon enhancing structures, single nanopores, and miniaturized fluid channels for lab-on-chip devices. In addition, the need to integrate “hard” materials with “soft” organic or biological molecules, together with the diversity of parts and techniques required to assemble biochips and biosensors, makes the fabrication extremely complex.^19^ The main fabrication challenges are attributed to creating biochemical patterns*/*structures at the desired location with precise design architectures (i.e., size, shape) and retention of their bioactivity. Furthermore, the need to obtain high sensitivity with low sample volumes has further driven the increasing exploration into micro- and nanoscale fabrication technologies.^20−22^

Lithographic approaches are widely used for biochips and biosensors production, as they are capable of producing complex micro/nanoscale structures with high-resolution topography, and can be adapted for the localized deposition of soft molecules while retaining their bioactivities.^19,23,24^ Even so, the harnessing of lithographic processes^25^ in biochip and biosensor fabrication remained in its infancy until the early 2000s mainly due to the limited accessibility of facilities and the complexity of operation. This situation has only changed more recently as a result of increased multidisciplinary collaborations between physical and life sciences, more investment in advanced R&D facilities, increased access to fabrication tools, and lower costs of production.

One of the main advantages of lithographic methods is their high spatial resolution. Feature sizes down to <10 nm are now possible, which cannot be realized by printing-based methods (Table 1). This capability permits a larger number of structures to be placed on a substrate (i.e., the packing density), thus reducing device size and possibly the amount of material required for fabrication. It also allows reductions in the amounts of reagents, analysis volumes, and processing times, decreasing the overall cost of the analysis, while maintaining high sensitivity.^26,27^ Furthermore, features on the micro/nanoscale more closely match the size regime of biological environments and are relevant for a range of applications involving cell and tissue interactions: for example, in devices for drug delivery or tissue engineering (see section on cell-based features).^22^ In addition, some lithographic techniques are able to deliver large patterned areas (>10 cm^2^), maintaining cost effectiveness and high throughput (>100 cm^2^ h^–1^) (Table 1), which are key considerations for manufacturability (see following section).^19,28,29^

**Table 1:** Overview of the Lithographic and Nonlithographic Methods and Highlights of Their Most Common Features

	capability				
method	interconnected structures	minimum feature size	advantages	limitations	ref
EBL/IBL	Yes	∼2 nm	High-resolution, Mask or mold not required	Not suitable for mass production	(40−43)
SPL	Yes	∼10 nm			
Photolithography	Yes	∼50 nm	Widely available, Well-controlled large area structures, Suitable for mass production	Expensive systems if nanoscale resolution is required, New mask needed when feature design change	(23,28,44)
Soft lithography	Yes	∼30 nm	No clean-room environment needed, Suitable for mass production, Suitable for rigid and flexible surfaces	Defects given by stamp deformation during the process, Master needed to generate the stamp, New master/stamp needed when feature design change	(45−47)
NIL	Yes	∼5 nm	Use of hard materials more resistant to deformation, Suitable for mass production, High resolution	New mold needed when feature design change	(24,48−50)
Printing	Yes	∼1 μm	Fully automated, Suitable for mass production	Low resolution	(36,51−53)
Self-assembly	No	∼2 nm	Inexpensive, High resolution	Random position of nanostructures (unless combined with top-down methods)	(20,44)

Nanoscale features can also be patterned by interference lithography that employs interference patterns of two or more light beams to create nanoscale areas of illumination.^30^ This method is capable of large area patterning and has been demonstrated for diagnostic device fabrication.^31^ However, its main limitation is that it is only capable of repetitive periodic patterns, not arbitrary (user-defined) patterns that are desirable for complex multicomponent devices. Very small nanoscale features, with dimensions between 2 and 10 nm, can also be obtained using “bottom-up” molecular self-assembly methods (e.g., by phase segregating block copolymers).^32−34^ However, the precise control of the resulting nanostructures’ orientation and positioning on the substrate, and its reproducibility, is not possible without also harnessing lithographic methods capable of arbitrary patterning to template the self-assembly.

This review aims to discuss the production of biochips and biosensors through micro- and nanolithographic methods that are capable of arbitrary patterning, are scalable to large areas (from 10 cm^2^ to m^2^),^28,29,35^ and are applicable for “soft” biological molecules or biologically compatible materials. For this purpose, the review is organized in terms of the type of material patterned on the device surface, and how the various lithographic methods are employed so that they enable the devices to fulfill their desired function. In particular, the review will emphasize the resolution, scalability, throughput, accuracy, and compatibility of the lithographic techniques for biomolecules, highlighting the advances brought by these improvements in the biochip and biosensor field. Due to the very large number of papers related to this topic, it is regretfully not possible within this concise review to discuss the many high-quality reports in the literature. Rather, a smaller subset of papers that exemplify innovative lithographic methods, especially when combined with advanced surface chemistry, will be discussed. It should also be noted that nonlithographic printing methods are also widely used for the fabrication of devices; readers interested in these methods are referred to other reviews on that topic.^36−39^

## Lithographic Technologies for Scalable Fabrication

The concept of lithography covers a very broad and varied family of surface fabrication methods. Thus, selection of the most appropriate method for a particular application requires the user to balance considerations of resolution, throughput, and cost.

**Figure 1:**
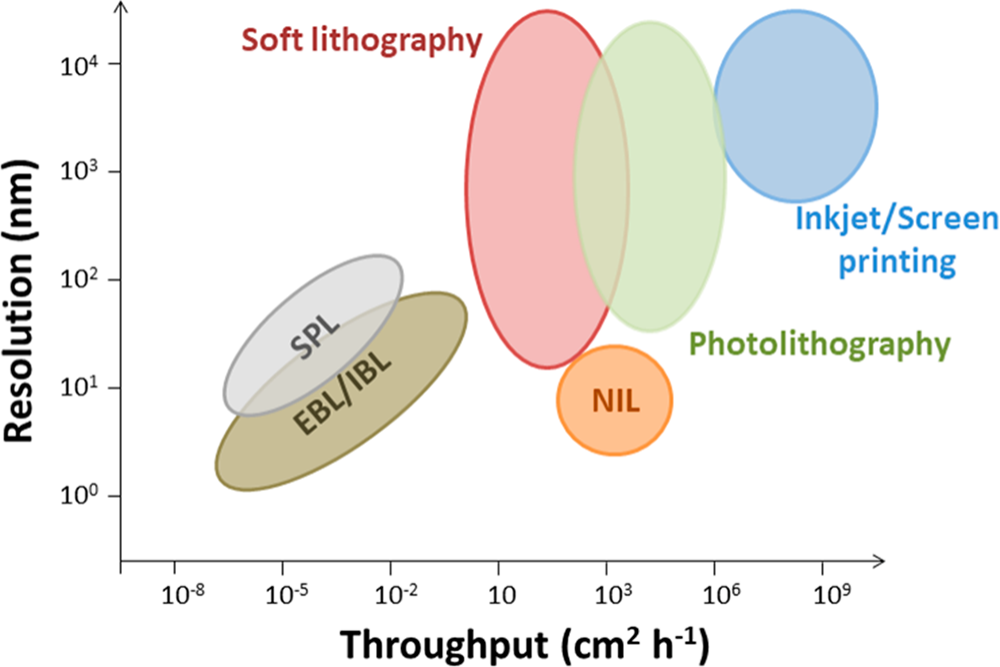
Graph of feature resolution versus fabrication throughput showing the relative location of various lithographic and printing processes.^23,29,40,43,58−60^

Of the commonly available techniques, the highest resolutions can be achieved by scanning probe lithography (SPL)^42,54−56^ and electron/ion beam lithography (EBL/IBL),^41,57^ which are capable of achieving features sizes of <10 nm. These techniques are “maskless”, in that they do not require a master, so offer the flexibility of turning over many different designs quickly. However, they are currently considered inappropriate for mass production because of the low fabrication throughput (quantified by area that can be patterned per unit time), typically achieving 10^–7^–10 cm^2^ h^–1^ (Figure 1).^23,29,40,43,58−60^

In order to achieve both high-volume production and high resolution at relatively low cost, other methods are necessary, such as photolithography,^61−63^ soft lithography,^58^ and nanoimprint lithography (NIL).^49,64^ These methods employ masks, stamps, and molds, respectively, to permit the simultaneous transfer and/or replication of identical patterns. These methods are capable of fabrication throughputs of >10 cm^2^ h^–1^ that are relevant for commercial production.

### Photolithography

Photolithography is the most mature and dominant method for micro- and nanofabrication in the semiconductor industry.^63^ In the photolithographic process, UV light is used to transfer a pattern defined on a photomask onto a photosensitive resist coated on the substrate. Over the years, several different types of photolithography have emerged, depending on the position of the mask relative to the photoresist layer and the UV light source.

**Figure 2:**
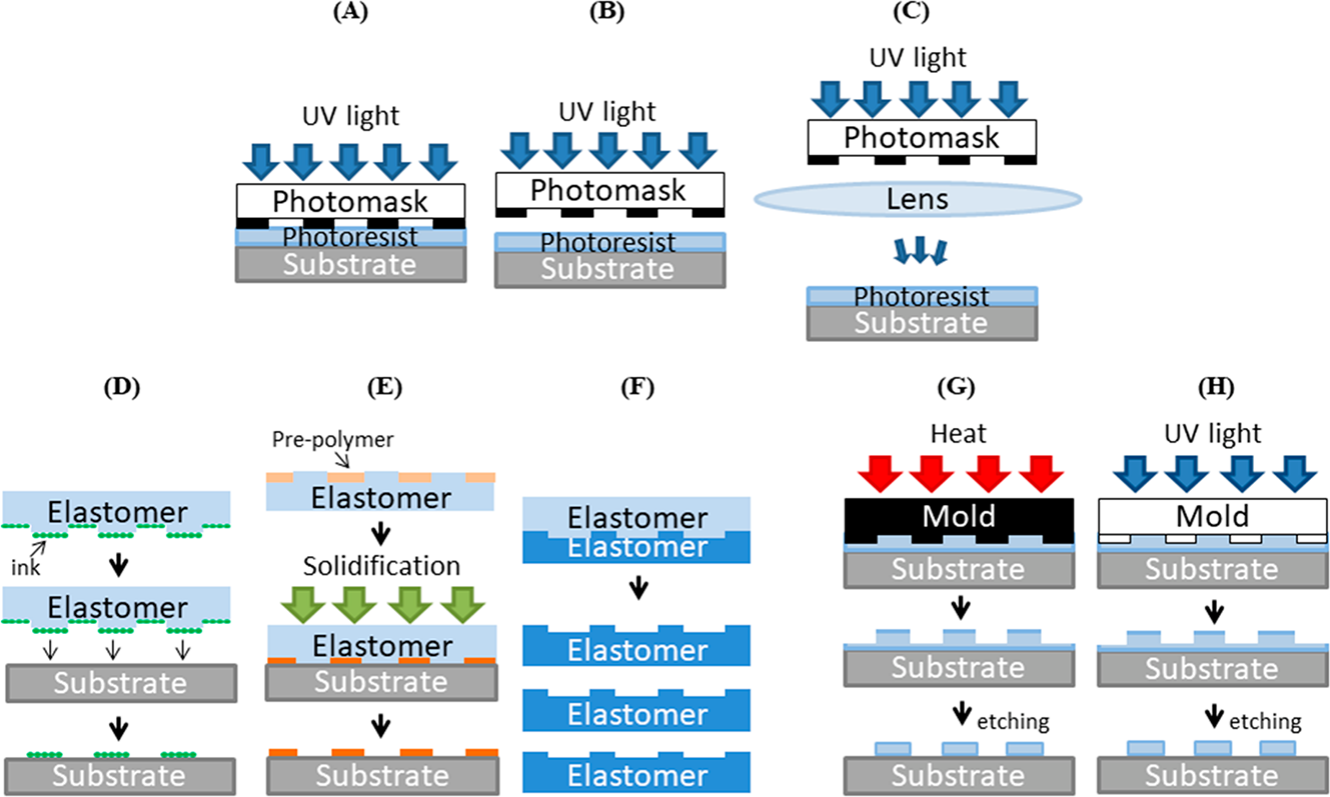
Schematic representation of large-area methods: (A) Contact, (B) proximity, and (C) projection photolithography. (D) Microcontact printing (μCP). (E) Microtransfer molding (μTM) and (F) replica molding (REM). (G) Thermal NIL and (H) UV-NIL.

Contact and proximity photolithography (Figure 2A,B) were the first methods that were demonstrated, and they remain widely used even at the current time, but they offer a relatively poor resolution of ∼1 μm.^23,65^ Improvements in resolution and edge contrast were then subsequently achieved with improved exposure tools (e.g., projection lens) and changes in irradiation wavelengths and photoresists, leading to projection photolithography, whereby a lens is employed to focus the mask patterns to smaller areas (Figure 2C).

The current state-of-the-art projection systems are able to reach resolutions down to a few tens of nanometers.^66^ On the other hand, projection photolithography requires complex systems that are less widely available, with higher running costs compared to contact and proximity photolithography.^65,67^ Regardless of the particular method, many decades of continuous process optimization in photolithography have now enabled the cost-effective patterning with high-throughput (∼10^4^ cm^2^ h^–1^).^42,67^ The maturity of the technology and its wide availability mean that it remains one of the main routes to the mass production of biodevices.^67^

### Soft Lithography

Soft lithography encompasses a broad range of related methods that generate patterned features using an elastomeric stamp, from which the desired pattern is transferred (Figure 2D,E). The elastomeric stamp, typically made of silicone (polydimethylsiloxane, PDMS), is produced by casting the prepolymer against a hard mold that itself is produced by photolithography or EBL. The stamp can be “inked” with the molecules of interest and stamping leads to the direct transfer of patterns of molecules defined by the topographical structure of the stamp.^22^

From this basic concept, several patterning processes have been developed.^58^ Microcontact printing (μCP) is the most mature soft lithography process where the elastomeric stamp is inked with the molecules that, when deposited on the substrate upon stamping, are able to form self-assembled monolayer (SAM) films (Figure 2D).^68−72^ In some examples, the stamp has been mounted on a microscope^5^ or a motorized system,^73^ leading to greater precision, repeatability, and higher throughput.

Another technique derived from μCP is microtransfer molding (μTM) (Figure 2E).^45,46,70,74^ In this approach, a liquid prepolymer is applied to the PDMS stamp, which is then brought into contact with a substrate. The molded prepolymer in the desired shape is then irradiated, heated, or treated with gelling agents to cure (solidify) the polymer. The elastomer is then lifted off to furnish the desired microstructures.

A variation on this approach is to use the soft stamps as a mold instead. In the replica molding (REM) method, a prepolymeric material (e.g., polyurethane, PDMS) is coated onto the stamp and cured (Figure 2F).^75^ Removal of the stamp thus yields the pattern of the solidified polymer, generating a negative copy of the stamp. In principle, this new copy can then be used as a stamp for other soft lithographic processes such as the previously mentioned μCP or μTM, or as a patterned substrate. Compared to μCP, both REM and μTM have the advantage of allowing three-dimensional (3D) topology transfer in a single step, whereas μCP only gives a molecular layer of ink. Moreover, higher pattern-transfer fidelity and resolutions are more easily achievable using REM.^76^

Soft lithographic methods can be implemented in a high-throughput manner (10^2^–10^3^ cm^2^ h^–1^)^77,78^ through the use of large reusable molds/stamps (cm^2^ areas) and automation of the process, and they can generate surface patterns with feature size down to 30 nm.^45^ Moreover, they are not subject to optical considerations related to diffraction and transparency that are limiting for photolithography.^70^ The use of a soft stamp also offers the advantages of allowing the transfer of patterns multiple times not only on rigid substrates, but also on flexible, curved, or soft surfaces. It also provides routes to complex patterns due to the isotropic mechanical deformation of PDMS.^70^ On the other hand, soft lithography is limited by the properties of PDMS. Specifically, patterns in the stamp may be distorted due to deformation of the elastomer.^71^ To address this issue, PDMS can be substituted with the stiffer poly(methyl methacrylate) (PMMA), giving a more rigid stamp that allows higher aspect ratios and fewer defects that may result from stamp deformation.^79^

Another limitation is that though producing the elastomeric stamp does not require specialized equipment or expensive materials, a silicon “master” mold is still needed to produce the stamp. Since this master is produced using photolithography or EBL, its cost and fabrication time need to be considered in the overall soft lithography process.^19^

### Nanoimprint Lithography

Contemporaneously with the development of soft lithography, in 1996 Chou et al.^80^ reported a stamp-based lithographic method which they termed nanoimprint lithography (NIL).^49^ Conceptually, NIL is based on replica molding, but instead of directly transferring the pattern to the substrate material, the stamp is pressed into a conformable resist material that covers the substrate. The resist is then cured and the stamp is removed. Any residual resist material can be removed by etching processes in order to complete the pattern transfer into the substrate (Figure 2G,H).^49^ Compared to REM, the mold used in NIL is typically of a hard material (e.g., Si) which is more resistant to deformation during the patterning process, and can thus achieve greater resolution and pattern fidelity. Indeed, the current state-of-the-art NIL systems have realized feature sizes down to 5 nm.^49^ Currently, two variations of NIL are widely used: thermal NIL and UV-NIL.^19,49,64,81^ These create patterns by deformation of imprint resist using heat or by curing a soft resist by UV light exposure, respectively. Resists used in thermal NIL are thermoplastic polymers formulated so that they can be spin-coated in a uniformly thick layer and be molded and demolded during the imprinting process. A commonly used resist for thermal NIL is PMMA, due to its low cost and availability in a wide range of molecular weights and polydispersities.^49^ UV-NIL instead uses low-viscosity UV-curable monomers as resists, which cross-link after the exposure to UV light to form a rigid polymer. A wide range of proprietary photocurable resist formulations are available, with the most widely used being Amonil and SU-8 (see examples below).^23^

The simultaneous use of thermal and UV curing has also been demonstrated.^16,50^ In this simultaneous thermal and UV NIL (STU-NIL), the applied heat softens the resist to give better conformation to the mold, followed by UV curing. STU-NIL offers the advantages of eliminating the need for cooling time prior to mold lifting, and minimizing deformation due to thermal expansion differences.^50^

Since these approaches were described, further development of NIL has been driven by the desire to increase their throughput. For example, UV-NIL has since been elaborated into step-and-flash imprint lithography (S-FIL), which is a step-and-repeat method.^82^ In addition, in 2008 a new approach called “roller NIL” was described where the mold was set on a cylinder that is rolled over the substrate to imprint patterns, which enables continuous “roll-to-roll” processing.^48^

As the infrastructure and equipment needed to implement NIL is relatively simple, it has begun to emerge as one of the most promising nanoscale manufacturing technologies^83^ for the mass production of low-cost, high-throughput (∼10^3^ cm^2^ h^–1^), and high-resolution micro/nanoscale patterns. Moreover, it can also be applied fabrication of complex 2D and 3D nanostructures.^17,84^

## Applications of Large-Scale Lithographic Technologies

### Lithography for Biochip Fabrication

As noted above, biochips are substrates where biomolecules (typically oligonucleotides or proteins) or whole cells are immobilized in an array format. These arrays can be fabricated by nonlithographic methods that provide micron-scale feature sizes. Mechanical printing, whereby a series of metal pins loaded with the sample solutions are used to deposit nanoliter volumes on the substrate, was one of the earliest methods described for the generation of microarrays. This is a mature technology that is still widely used, as the equipment needed to perform printing in this way is readily available and well-optimized for this purpose.^51,52,85^ Subsequently, deposition methods based on inkjet printing have also been developed to allow the noncontact printing of a variety of materials including oligonucleotides,^86^ proteins,^87^ biomaterials,^88,89^ and even live cells.^90^

However, these “wet” methods suffer from a number of drawbacks that can limit the quality of the printed features and the density of features per unit surface area. First, these methods require a carrier solvent that evaporates after printing, which can result in irregular feature shape and uniformity. Another drawback of inkjet printing is that the distance between the spots on the array surface must be large (tens of micrometers) to prevent the droplets from being ejected by the inkjet printer from coalescing. Moreover, the resolution of each feature is limited by the printhead nozzle diameter, leading to a minimum feature size of ∼1 μm.^19,91,92^ As a consequence, printed microarrays can achieve only relatively low feature densities of <30,000 features per glass slide (∼2.5 × 7.5 cm^2^).^51^ In comparison, the superior spatial resolution offered by lithographic methods has now been shown to be capable of arrays with densities of >500,000 features per glass slide (see below).^61,93^

### Lithography of Oligonucleotide Features

DNA or RNA microarrays, often colloquially referred to as “DNA or RNA chips”, consist of substrates upon which thousands of micrometer-scale oligonucleotide features are deposited. Each feature on the chip surface can either be a different oligonucleotide sequence or the same sequence. In both cases, the identity of the sequence and its location on the substrate are known; i.e., each location on the chip corresponds to a known sequence. These chips can be used in a variety of ways to determine the genetic, transcriptomic, or proteomic profile of a biological sample, primarily by using the deposited oligonucleotide to capture its complementary oligonucleotide from the sample or by the *in situ* translation of the gene to the corresponding protein.^51,94^ From the perspective of fabrication, the main technical issues are the generation of high densities of features whereby each feature consists of only one sequence. Furthermore, those nucleotides must be deposited in a manner that maintains their biological function.

Here, two large-area lithographic approaches can be envisaged: stepwise synthesis of the oligonucleotide strands (one nucleotide at a time) on the chip, and the one-step printing of complete strands that have been synthesized elsewhere.

#### Oligonucleotide Features Fabricated Using Photolithography

Currently, photolithography is the most widely used method for the stepwise synthesis of DNA directly onto the individual microarray features, since it is possible to address specific locations on their surface, and to incorporate multiple exposure steps to build up the desired nucleotide sequence. This process has been exploited by Affymetrix (now Thermo Fisher Scientific),^95^ which has demonstrated high-density oligonucleotide microarray with ∼25-mer DNA strands. This technology forms the basis of their commercial products and has since been automated to give a high level of reproducibility,^96^ with a typical 1.28 cm^2^ Affymetrix microarray containing more than 1.4 million features. This array density compares favorably to microarrays produced by Agilent based on stepwise oligonucleotide synthesis by an inkjet printing that can only achieve ∼25,000 features on 18.75 cm^2^.^97^

Two limiting characteristics of the current stepwise photolithographic synthesis of oligonucleotide strands are that (i) the chemistry is not sufficiently robust for the production of sequences that are longer than ∼25 nucleotides, while inkjet printed microarrays allows the formation of up to 60-mer strands; and (ii) it employs contact or proximity photolithography (Figure 2B) which provides a feature size of ≥0.5 μm.^95,98,99^

**Figure 3:**
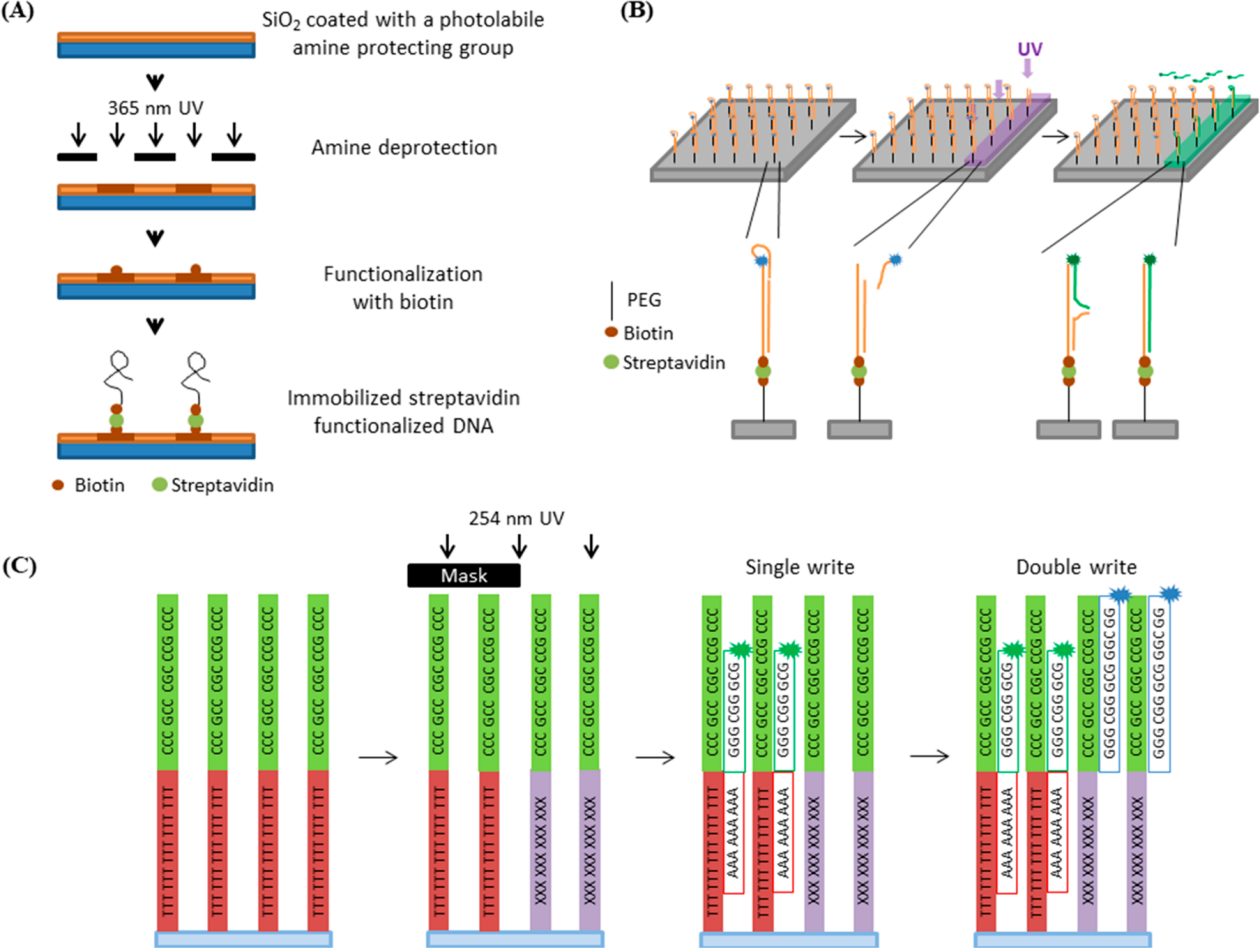
(A) Schematic representation of the photolithographic process and capture of long DNA strands. In this approach, the surface presenting amines blocked with a photolabile protecting group are subjected to photolithography, which removes the protecting group. The exposed areas are then functionalized with biotin. Subsequent immobilization of streptavidin then allows the capture of biotinylated DNA strands. Redrawn by the authors from ref (100). (B) Scheme of one-step photolithography on immobilized hairpin DNA loops containing a photocleavable linker. Light exposure results in the release of the hairpin, enabling the hybridization and capture of an incoming DNA strand. Redrawn by the authors from ref (14). (C) Schematic diagram of the DNA double-write process. In this approach, the exposed thymine bases form dimers (marked as X) which prevent the hybridization of probes containing adenine, leading to the first “write” step. The second write step is performed by hybridizing a different complementary DNA sequence, which does not include adenine, on the exposed areas. Redrawn by the authors from ref (99).

In order to address the first issue, more recent development has worked toward using a single-step photolithography that produces exposed areas for the capture of longer DNA strands sourced from conventional methods. In one example, fabrication begins with a SiO_2_ substrate coated with an organosilane film that presents an amine protected with a photocleavable *o*-nitrobenzyl group. Photoexposure results in the cleavage of the protecting group to reveal the amine, which is then functionalized with biotin. Incubation with streptavidin followed by biotinylated DNA strands then results in the immobilization of the DNA onto the exposed areas (Figure 3A).^100^ This approach enables the fabrication of DNA chips with long strands of up to ∼2000 nucleotides. These long strands can further be subjected to *in vitro* translation, to produce the protein that is encoded by the DNA, thus enabling the creation of protein microarrays from the corresponding DNA microarray. Using this approach, the fabrication of a simple two-stage biomolecular signaling pathway was demonstrated, where a protein produced at one location diffuses to regulate the synthesis of another protein at a second location, proving the concept of on-chip biochemical circuits.^100^

Another single-step photolithographic method has been recently presented that uses a surface presenting DNA hairpin loops containing a photocleavable linker (Figure 3B).^14^ In this case, light exposure cleaves the linker and opens the hairpin to enable the hybridization (and thus capture) of complementary DNA strands subsequently pipetted on the surface. In this approach, all the steps were performed in physiologically compatible conditions, ensuring the compatibility with a wide range of biomolecular agents. As with the previous method, the immobilized DNA strands could also be translated to their corresponding protein.

These single-step methods, however, all rely on the capture of the incoming DNA by various noncovalent interactions (e.g., biotin–streptavidin interaction, DNA hybridization) that are relatively weak. In order to produce robust DNA immobilization, cinnamate-modified linkers can be employed, whereby UV light exposure results in the covalent cross-linking of hybridized DNA strands.^98^

In all these one-step methods, a synthetic photocleavable protecting group or linker is necessary, which complicates the design of the immobilization process and cost. More recently, a method has been reported that achieves selective DNA hybridization using standard DNA strands, without the need for nonbiological DNA cross-linkers or modifications.^99^ This method relies on the photolytic dimerization of adjacent thymidine nucleotides upon UV irradiation, which prevents hybridization of an otherwise complementary strand. Thus, on surfaces bearing a short DNA strand that contains a polythymidine sequence, photolithography results in patterns on which there is no capture of the incoming strand pipetted on the surface after the lithographic step (Figure 3C). This results in “negative tone” lithography (i.e., DNA capture occurs in the unexposed area), which contrasts with the other photolithographic method discussed so far in this section. In addition, further UV patterning can be carried out in both the UV exposed and nonexposed areas by designing different complementary DNA sequences, which makes this approach a “double-write” process (i.e., two sequential immobilizations on the same area).

In regard to RNA microarrays, these were historically prepared by the printing of RNA sourced from conventional methods, or from the *in situ* transcription of the corresponding DNA microarrays.^85^ However, the relative chemical instability of RNA compared to DNA has meant that these methods for microarray production were generally inefficient. Stepwise RNA synthesis is also more chemically complex than the corresponding process for DNA, and it has only been within the last 2–3 years that stepwise photolithographic preparation of RNA microarrays has reached efficiencies comparable to their DNA counterparts.^61,101^ The most recent reports have demonstrated RNA strands of up to 30 nucleotides in length, in 14 × 14 μm features with a maximum achievable density of 786,432 features per array.^61^

### Oligonucleotide Features Fabricated Using μCP

Where large arrays of features are required consisting of identical DNA sequences, stamp-based methods can offer simple and low-cost alternatives to photolithography. It is also possible to achieve submicrometer resolution by μCP.^102^ μCP has the advantage of printing homogeneous and thin films compared to droplet or ink jet printing methods, offering better resolutions and using lower quantities of DNA.^70,73,102^ Indeed, it has been demonstrated that DNA strands printed by μCP enables a greater amount of the immobilized DNA to be accessible by the incoming test samples. This effect arises from the fact that μCP is a “dry” (solvent free) method that gives rise to better organized and densely packed molecules.^102^ Another important advantage of μCP is that since only very small amounts of molecules are deposited with each contact, a single stamp inked once can be used for multiple printing steps, yielding uniform surfaces with excellent edge definitions.^102,103^

However, in this method of printing, the deposition of the ink molecules is very dependent on the intermolecular interactions between the ink, stamp, and surface. The attractive interactions between the stamp and the negatively charged and highly polar DNA must be sufficient to enable the DNA to be spread onto the stamp during inking, but not bound so strongly that the DNA is not transferred to the substrate upon contact printing (i.e., the adhesion of the DNA to the substrate must be stronger than to the stamp). In an example where the stamp surface can be tailored for DNA printing, the stamp was silanized with (aminopropyl)triethoxysilane to expose positive charges on its surface, before incubating it with DNA solution.^103^ This DNA-inked stamp was then shown to be able to deposit the DNA onto positively charged amine glass slides, with resolutions down to 1 μm.

**Figure 4:**
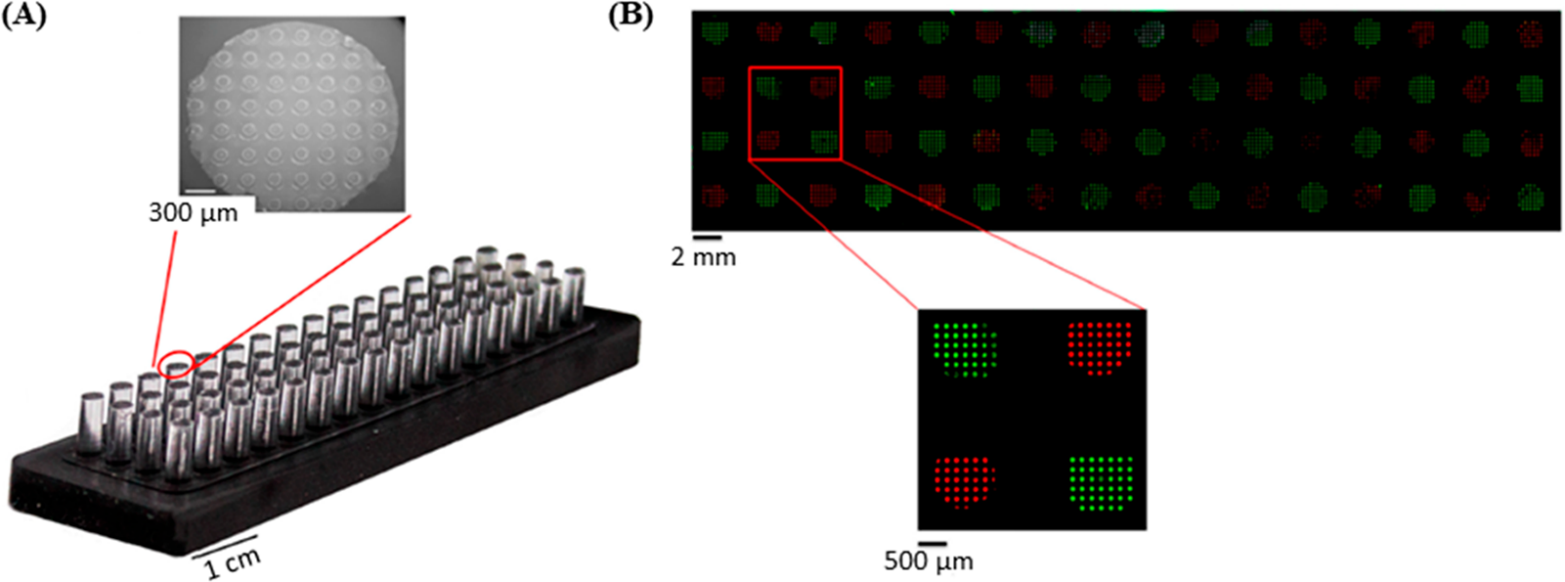
(A) Photographs of a printing device with an array of 64 μCP stamps, each with an array of 160 μm features. (B) Fluorescence microscopy image of multiplexed DNA printing, showing each individual stamp depositing a different ink. Figure adapted from ref (73). CC BY 4.0.

There has also been a demonstration where μCP was used to fabricate large arrays of features with different DNA sequences by using a stamp made of 64 pillars, each mounted with 50 circular micropatterns (spots) of 160 μm diameter at 320 μm pitch (Figure 4). The stamp was inked by immersion in a microtiter plate, where each pillar fits one well of the microplate and can potentially be inked with a different DNA sample by varying the DNA content of each single well of the plate.^73^ Thus far, a resolution of only 160 μm (feature diameter) has been reported, but it is anticipated that greater resolution can be achieved with further optimization.

### Oligonucleotide Features Fabricated Using REM

**Figure 5:**
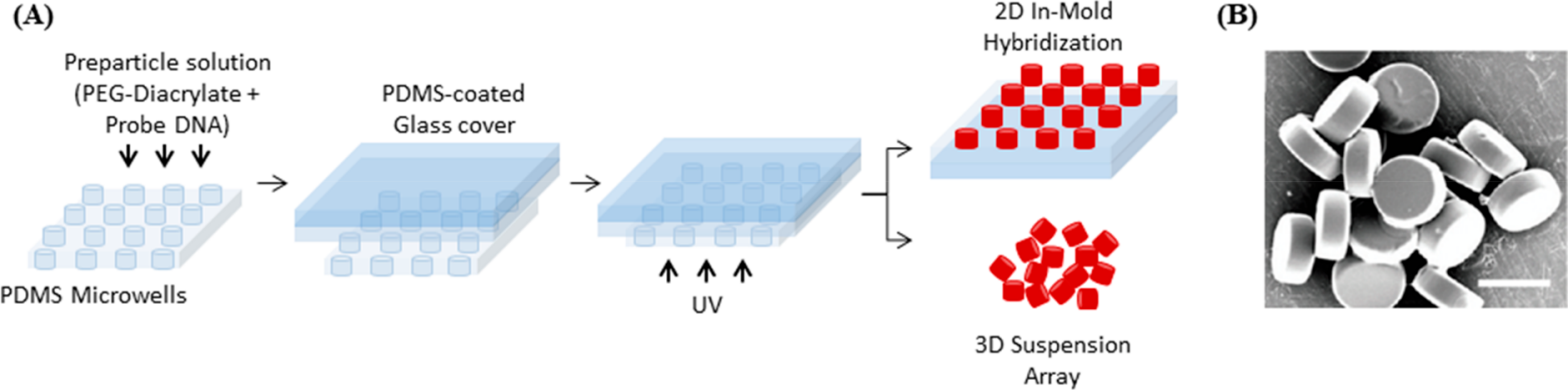
(A) Scheme of DNA microfeature fabrication via REM. The PDMS microwells are filled with the prepolymer solution of PEG-diacylate and DNA, covered with a PDMS-coated glass slide, and UV light is used to trigger the polymerization. This approach can be used to generate either an array of 2D structures immobilized on the substrate or 3D suspended microparticles. Redrawn by the authors from ref (104). (B) Scanning electron microscopy (SEM) image showing shape and dimension of DNA-conjugated hydrogel microdisks fabricated via REM. Scale bar represents 50 μm. Reproduced from ref (104). Copyright 2010 American Chemical Society.

In one notable example where REM was applied for the fabrication of biomolecular arrays, it was used to mold arrays of DNA-conjugated polyacrylamide hydrogel features.^104^ Here, REM was used in combination with UV photopolymerization: a prepolymer solution, made of ssDNA, poly(ethylene glycol)(PEG)-diacrylate, and a photoinitiator are added into the PDMS microwells covered with a PDMS-coated glass cover, followed by UV exposure. Using this method, each feature could be molded to form 3D structures such as discs, cubes, and prisms, with dimensions of ∼50 μm. The method also enabled the generation of suspensions of these structures by lift-off from the substrate post-lithography (Figure 5). In all cases, it was demonstrated that the conjugated DNA strand retained its ability to hybridize with complementary strands. The authors proposed that these microstructures could be used for applications in high-throughput biosensing with low sample volume and rapid detection.

### Oligonucleotide Features Fabricated Using NIL

In cases where nanometer resolution of features is required, NIL can be employed. The main disadvantage of applying NIL for this purpose is that since it is a method for molding stiff materials, it does not directly deliver the biomolecules, and a multistep process is required to generate the final DNA-presenting features. Nevertheless, several examples harnessing NIL for DNA arrays have been reported.

**Figure 6:**
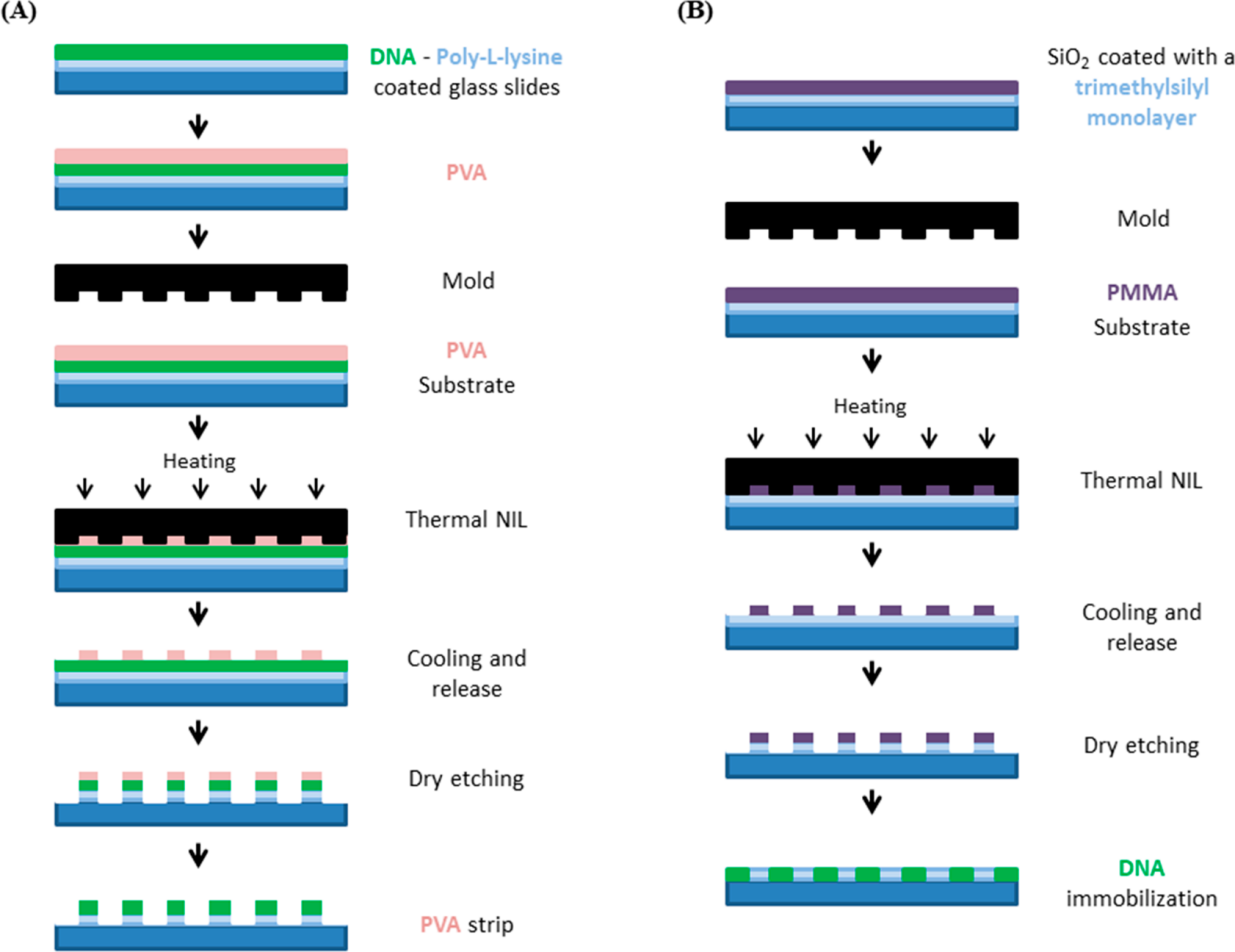
Illustrative scheme of NIL for the generation of (A) negative and (B) positive tone DNA features. Redrawn by the authors from refs (105) and (106).

In the earliest report using NIL (Figure 6A),^105^ DNA was first coated over the entire surface of a substrate followed by a coat of poly(vinyl alcohol) (PVA). Thermal NIL was then performed on the PVA layer to generate the desired pattern, which then acted as a resist for an oxygen plasma etching step that removed the residual PVA and underlying DNA from the stamped areas. Finally, the remaining water-soluble PVA was then removed by washing to furnish the final substrate with patterned DNA. Using this method, negative tone features (i.e., features in areas not contacted by the NIL stamp) with line widths of 700 nm and space widths of as low as 800 nm were demonstrated. Despite the multiple processing steps that involved heating and plasma exposure, it was found that the DNA features remained apparently intact and could be selectively visualized by subsequent labeling with a DNA-intercalating fluorescent dye.

In order to generate positive tone DNA features, an alternative approach is required whereby NIL is used to define surface features that are subsequently able to capture incoming DNA. In the reported example, a silicon dioxide substrate was coated with a hydrophobic trimethylsilyl monolayer, upon which thermal NIL was performed using PMMA as the resist (Figure 6B).^106^ Subsequent etching then resulted in the removal of the trimethylsilyl layer, revealing the underlying (more polar) silicon dioxide substrate with the pattern defined by the NIL stamp. Finally, immersion with a solution of DNA resulted in the DNA adsorbing to the silicon dioxide features. Using this process, patterns of 100 nm with space widths of 500 nm were achieved.

#### Comparative Analysis of Fabrication Methods for Oligonucleotide Features

Overall, each of the large-scale lithographic methods for oligonucleotide feature fabrication provides several distinctive capabilities. Photolithography is capable of producing arrays of individually addressable features (i.e., each bearing a different biomolecule), with the current commercial examples containing ∼25,000 unique sequences on a single chip.^107^ However, this method requires relatively high cost and specialized equipment that is not widely available outside dedicated microarray manufacturers. Currently, the limitations of conventional far-field optics mean that features below ∼200 nm are not readily achievable by this lithographic method, though future widespread implementation of more sophisticated projection photolithography techniques (e.g., extreme ultraviolet interference lithography) may enable sub-100 nm resolutions while maintaining high throughput.

The μCP methods, on the other hand, have resolutions in the micron range. Indeed, there are now credible examples of parallelization and automation of μCP to enable the printing of arrays presenting multiple DNA sequences on a single chip.^73^ Although its resolution is inferior to that offered by photolithography, the low cost of implementation, together with the fact that the PDMS stamp can be used for multiple times without being re-inked (thus conserving DNA material), are significant advantages.^7,102^

NIL-based fabrication processes could offer a low-cost route to the fabrication of high-resolution (down to 100 nm readily achievable) and high-density oligonucleotide arrays. However, many technical challenges remain to be addressed in order to achieve the scale of parallelization that has been demonstrated with photolithography: for example, streamlining the multistep processes needed to generate the final oligonucleotide microarray, and developing new strategies to enable the fabrication of arrays bearing multiple sequences on a single chip.

### Lithography of Protein and Short Peptide Features

By analogy to the oligonucleotide microarrays, protein and peptide microarrays are devices where these biomolecules are site-selectively immobilized onto a solid surface. “Peptides arrays” presenting short sequences of amino acid residues (typically 3–12 residues)^108−110^ are mostly used to study cell behavior, such as cell differentiation or adhesion, since cell surface receptors primarily recognize only small areas on extracellular matrix proteins. “Protein arrays” that present whole protein molecules have found a wider range of applications including protein interaction studies, immune profiling, vaccine development, biomarker discovery, and clinical diagnostics. As a result, enzyme and antibody microarrays are the most commonly reported in the scientific literature.^19,22^ In addition, there are now emerging applications for protein chips in the discovery of biomaterials compatible with drug release, medical implants, tissue engineering, and regenerative medicine.^111−113^

From the perspective of their fabrication, in addition to the considerations that apply to oligonucleotide chips discussed above, the process involved must be able to maintain the correct protein folding and hence their biochemical function.^52^ A widely adopted method for the production of functional protein microarrays involves *in situ* transcription and translation of DNA arrays, which was first reported in 2004 by LaBaer et al. and termed “nucleic acid programmable protein arrays” (NAPPA).^11,114^ In NAPPA, the translated proteins are captured under physiological conditions (see following section), and the translation is carried out only immediately prior to analysis, thus avoiding the loss of protein integrity or activity, while offering reproducibility and throughput.^115−122^ Other approaches involve the deposition onto the chip surface of short peptides or proteins produced from standard methods (see following sections).

In all cases, the surfaces of these arrays must also be engineered so that they avoid nonspecific protein adsorption that may interfere with subsequent application or analysis.

### Protein and Short Peptide Features Fabricated Using Photolithography

In conventional NAPPA, one limitation has been the relatively low densities of protein features that are achievable (>600 μm between each protein “spot”). This relatively large interfeature distance is needed since *in vitro* transcription and translation generate mRNA and protein in solution, which can diffuse some distance before being immobilized on the substrate. Thus, in order to achieve higher densities, modification of the standard planar substrates is necessary. For example, contact photolithography has been employed for the fabrication of arrays of “nanowells” (250 μm diameter, 75 μm depth) so that the reagents for transcription and translation could be confined within the wells by inkjet printing.^12,123^ Arrays of these nanowells thus enabled the distance between each feature to be reduced to 125 μm, allowing a 4-fold increase in array density.

**Figure 7:**
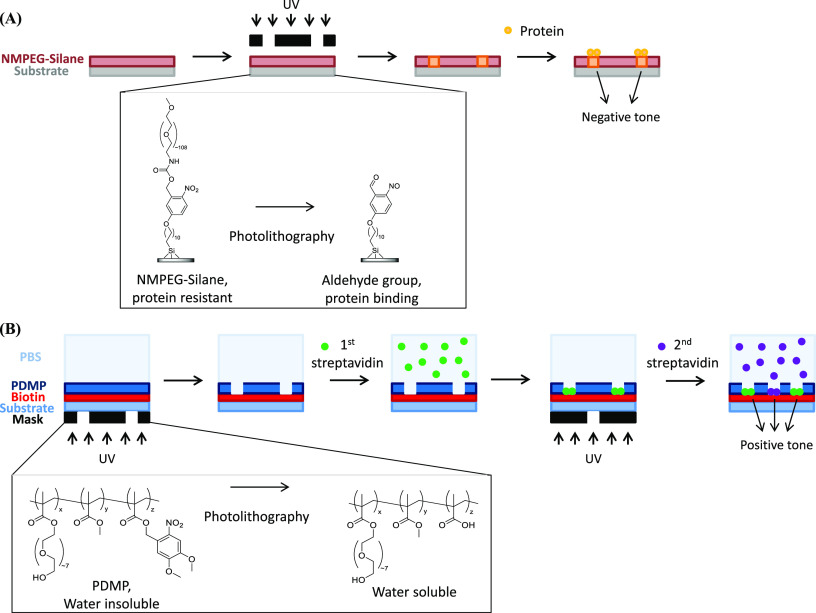
Schematic representations of the photolithographic generation of protein features. (A) For negative tone photolithography, the substrate is functionalized with PEG chains that linked to the substrate via a *o*-nitrobenzyl photocleavable linker. Photoirradiation results in the cleavage of the linker to reveal an aldehyde that is used for subsequent protein immobilization by imine bond formation. The linkage can then be rendered irreversible by reduction of the imine under mild conditions. (B) For positive tone features, PDMP is photoirradiated to generate a water-soluble byproduct, which can be washed away to expose the underlying biotin-functionalized substrate for protein binding. Redrawn by the authors from (A) ref (124) and (B) ref (125).

In order to prevent nonspecific protein adsorption (“biofouling”) in the unprinted areas, photolithography can be employed in conjunction with coatings that are resistant to protein adsorption, whereby photoirradiation results in the removal of the coating and/or generation of a functional group that enables immobilization of the protein (Figure 7A). This process, repeated on different areas of the surface, can potentially lead to the immobilization of different proteins on the same microarray. In an early example of this approach, the substrate was coated with an organosilane film presenting hydrophilic PEG chains that resisted nonspecific protein adsorption.^124^ These PEG chains were attached to the underlying substrate with *o*-nitrobenzyl photocleavable linkers, so that cleavage of the linker resulted in the loss of the PEG chain and the generation of an aldehyde group. The incoming protein could then be immobilized by imine formation with these aldehydes on the exposed areas, thus generating negative tone features (i.e., biomolecules are present in the areas that were photoexposed). Using this type of surface chemistry and simple proximity photolithography equipment, protein lines of ∼1 μm in width and ∼2 μm apart were readily achieved.

In addition to negative tone features, photolithographic methods can generate positive tone features overall (i.e., biomolecule patterns are present in the areas that are not exposed) by using materials that are resistant to protein adsorption, but can be degraded and removed by photoexposure to reveal the underlying substrate for protein immobilization.^125,126^ Examples of bioresistant materials that have been used to generate positive tone features include poly(2,2-dimethoxy nitrobenzyl methacrylate-*r*-methyl methacrylate-*r*-poly(ethylene glycol) methacrylate) (PDMP) and PVA, both of which generate water-soluble byproducts upon photodegradation. In the former example, PDMP was used as the resist above a biotin-functionalized glass substrate, thus revealing the biotin after photolithography and washing.^125^ The strong biotin–streptavidin interaction was then exploited to immobilize proteins in the exposed areas (Figure 7B). Here, using a simple microscope projection photolithography system, multiple exposures were demonstrated that enabled immobilization different proteins after each exposure, as well as facile fabrication of features with resolution down to 1 μm.

**Figure 8:**
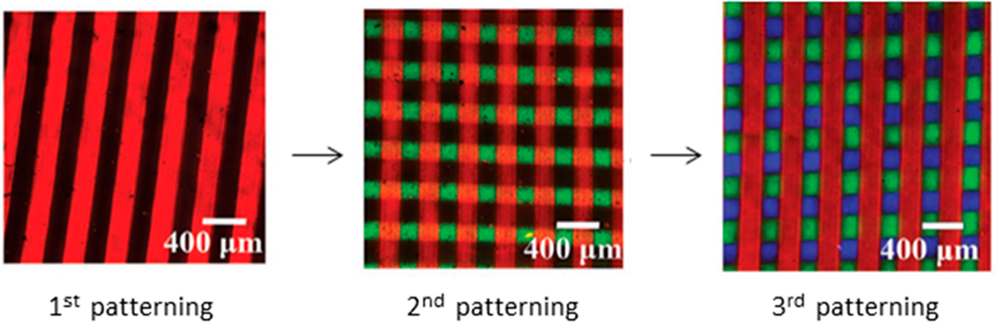
Epifluorescence microscopy images of multiprotein patterns produced using photolithography. Red lines: Rhodamine-labeled bovine serum albumin; green squares: fluorescein-labeled hydrogel; blue squares: Alexafluor-405-labeled avidin. Figure adapted with permission from ref (127). Copyright 2018 John Wiley and Sons.

The concept of using photocleavable linkers to control the release of groups for subsequent protein immobilization is not limited to thin film materials. In another example, the same *o*-nitrobenzyl linker was used in a 1-mm-thick polyacrylate hydrogel layer, where proximity lithography was exploited to create patterns within the hydrogel that was impregnated with a variety of proteins with lateral resolutions of 100–200 μm.^127^ The ability to perform lithography within the volume of a hydrogel material is particularly useful for biochips that are intended for interfacing with cells, since these hydrogels mimic the physicochemical properties of the extracellular matrix.^128^ Moreover, it is possible to use multiple exposures and protein immobilization steps to generate complex designs where different areas present different proteins (Figure 8).

Recently, direct patterning of protein films was obtained using chemically modified proteins without significant change in protein structure and function, representing a new strategy for the scalable photolithography of structural proteins.^112,129−131^ Here, films of silk fibroin or wool keratin functionalized with methacrylate groups were used as the photopolymerizable resist material. Contact photolithography enabled the generation of 2D microstructures with sizes down to 1.5 μm over macroscale (cm^2^) areas. These silk fibroin-based “photoresists” offer high mechanical strength and biocompatibility,^129,130^ but had relatively poor repeatability and low pattern contrast due to the wide molecular weight distribution of the naturally extracted protein. However, subsequent use of only the refined fibroin light chain showed improved pattern resolutions and contrast.^112^ More recently, the use of wool keratin for the same application has also been reported.^131^ This material had similar lithographic characteristics to silk fibroin, but the unpolymerized proteins can be dissolved in water (silk fibroin requires the use of toxic solvents, such as hexafluoro-2-propanol or trifluoroethanol), making its processing completely water-based, making it cheaper to use and more environmentally friendly. These silk fibroin and wool keratin patterns were subsequently applied to study cell behavior, such as the spatial guidance of fetal neural stems cells, and tissue engineering.

As noted above, cell culture substrates that have surfaces presenting microscale features of a single short peptide have been widely used to investigate cell responses to their microenvironment and to culture cells for therapeutic purposes (e.g., replacement cells in regenerative medicine). Such peptide-patterned substrates are readily fabricated by photolithography. Conceptually, the approach is similar to the other biomolecular arrays mentioned above, whereby light exposure is used to direct the subsequent immobilization of the peptides to specific locations on the surface. In one example, RGD and BMP peptides that promote cell adhesion and differentiation, respectively, have been micropatterned on glass substrates.^132^ These substrates were fabricated by first functionalizing the surface so that it presented maleimide groups, upon which was coated a positive photoresist. Removal of the resist in the photoexposed areas revealed the maleimide groups that were used to capture thiol-presenting peptides. These substrates were then used to investigate how the patterned substrates resulted in altered human mesenchymal stem cell (hMSC) differentiation, in comparison to the unpatterned surface.

Large-area lithographic methods are particularly relevant for this type of experiment, since relatively large areas must be fabricated in order to culture sufficient numbers of cells for statistical analysis and/or downstream biochemical analysis by standard methods (e.g., Western blotting, sequencing). Furthermore, the ability to fabricate arbitrary patterns is also beneficial in order to investigate the effect of different shapes, aspect ratios, or sizes of the features on cell behavior. For example, in the same study it was found that triangular and square BMP features of 10 and 7 μm and aspect ratios of 1 and 0.7, respectively, enhances the osteogenic differentiation of hMSCs in the absence of any induction media, compared to randomly distributed peptides on unpatterned surfaces.

### Protein Features Fabricated Using μCP

Where high-throughput yet low-cost printing is required at medium resolution (10^–2^ to 10^–7^ m), μCP is an attractive option. The avoidance of harsh conditions such as UV light (in photolithography) or high temperatures and pressures (in NIL) is also desirable, as it reduces the likelihood of protein denaturation during the lithography process.^5,7^

A widely used application of μCP is in the printing of proteins onto substrates that are used in cell biology studies. As an example, 12-mm-diameter glass substrates printed with proteins were used in neuronal cell development studies.^133^ Here, the generality of the method was demonstrated by the printing of a range of signaling proteins including Semaphorin 3A, nerve growth factor (NGF), brain-derived neurotrophic factor (BDNF), and Netrin-1. 50-μm-wide stripes spaced 50 μm apart were obtained, which were reported as optimal dimensions for the subsequent demonstration of how these patterns affected the biochemistry of the cultured neurons.

**Figure 9:**
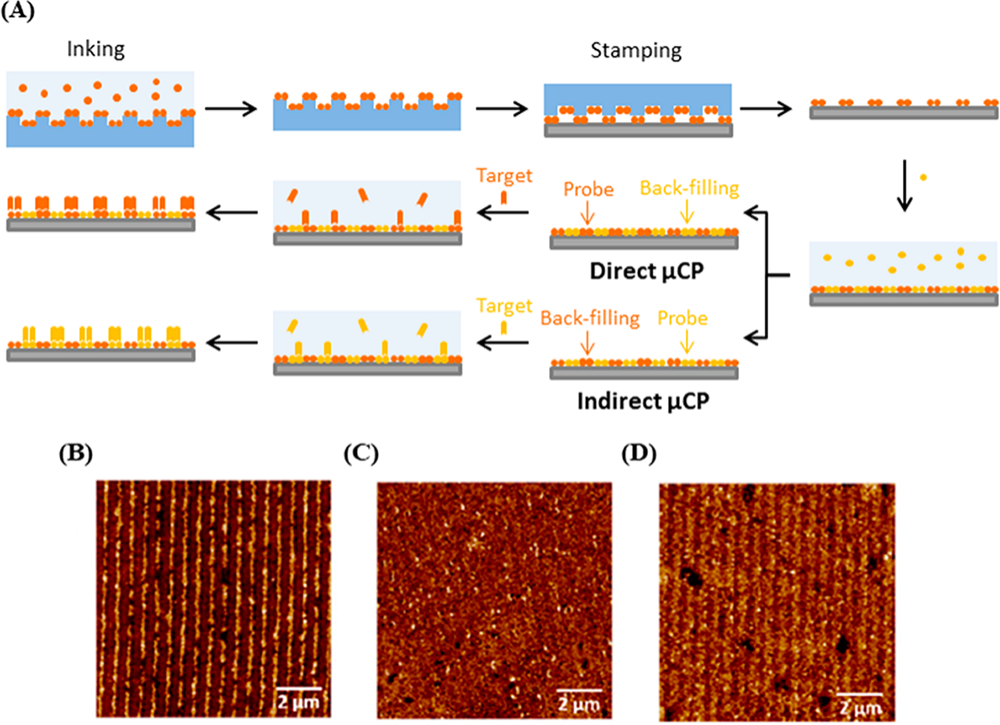
(A) Scheme of the fabrication process using direct and indirect μCP. The μCP stamp (blue) was used to print the first protein (red) onto the substrate (gray). In direct μCP, the first protein (red) is the probe, bound by the target, while the second protein (yellow) is the backfilling protein which is physisorbed onto the gaps. In indirect μCP, the first protein (red) is the backfilling, while the second protein (yellow) physisorbed is the probe, bound by the target. Redrawn by the authors from ref (72). (B,C,D) AFM topographic images of the different stages of indirect μCP. In (B), initial BSA patterning was indicated by raised features (brighter contrast). Subsequent backfilling with probe anti-IgG (C) resulted in the average height difference decrease, which increases again (D) after the incubation with target IgG, indicating probe–target interaction. Figures adapted from ref (72). CC BY 4.0.

Apart from direct deposition of the protein of interest, μCP can also be used to print templates from which a second protein can then be used to backfill the unprinted areas to give “indirect” fabrication of protein features. Both direct and indirect strategies have been demonstrated in the fabrication of glass diffraction gratings for biosensing, where the grating consisted of stripes of antibody features 140 nm in width at a 555 nm pitch.^72^ Here, the antibodies were printed either directly by μCP or indirectly by first printing bovine serum albumin by μCP and coating the unprinted areas with the antibody (Figure 9). These methods therefore offered contrasting positive and negative tone patterning. An additional advantage of indirect patterning is that it avoids exposing the protein of interest to the stresses associated with printing.

### Protein and Short Peptide Features Fabricated Using NIL

Since NIL is not a method that involves the transfer of materials, fabrication strategies for proteins thus rely on the formation of template patterns by NIL followed by the deposition of the protein of interest onto the patterned areas. Typically, NIL is used to generate patterns in an etch resist. Etching of the substrate under the patterned areas then allows for the selective functionalization and protein immobilization via a range of bioconjugate chemistries.^111,134,135^

Compared to photolithography and other soft lithographic methods, NIL has the major advantage of reaching higher resolutions and densities. In one example, NIL was exploited to generate an actin–myosin motor system whereby myosin immobilized on the substrate transported actin filaments traveling above it. To enable unidirectional movement of actin, it was necessary to fabricate narrow myosin tracks (<300 nm), and thus, UV-NIL was used to mold 200 nm channels in a TU-7 resist, with the base of each channel exposing the underlying silicon dioxide substrate.^136^ Silanization of the exposed substrate then enabled myosin to be selectively immobilized in these channels. NIL was found to be particularly advantageous in this application, since the patterned TU-7 resist showed an improved actin sliding velocity compared to channels made using the CSAR 62 resist patterned through e-beam lithography.

In the most extreme example, sub-10 nm peptide features with pitches down to 40 nm were produced, which are relevant in order to investigate molecular-scale protein–protein and protein–cell interactions.^111^ Here, thermal NIL is used as part of the process to generate arrays of AuPd alloy “nanodots” (on either silicon or glass substrates) that template the attachment of proteins. The desired patterns were generated by NIL of PMMA. Ti is then deposited at a tilt of 45°, which leads to a metal mask whose features are narrower than those originally defined by NIL. Subsequently, the surface is treated with oxygen plasma to remove the residual resist, and AuPd is then deposited by thermal evaporation in patterned areas. Functionalization by a mixed alkylthiol SAM of ethylene-glycolundecylthiol and biotinylated ethylene-glycol-undecylthiol then enabled biomolecule immobilization via an avidin linkage. This example is particularly significant, as it demonstrates a route to features that are smaller than can typically be achieved by routine NIL.

In the above examples, NIL has been used to pattern a conformable etch resist material, which then serves as a template for the subsequent patterning of the protein. However, an alternative application of NIL is to use a protein film itself as the conformable material, thus directly producing topographic features on the protein film. One study has shown that BSA, hemoglobin, and lysozyme could be patterned on silicon wafers with thermal NIL,^137^ to generate protein features with widths of 303 nm, periods of 606 nm, and groove depths of 190 nm. These topographical protein films can then be used in cell biology studies. This direct use of protein mixtures as a conformable material suggests a route to more straightforward fabrication processes. However, it remains unclear if this approach is readily applicable to a wider range of proteins since the conditions employed by NIL may result in the denaturation of more delicate proteins.

### Comparative Analysis of Fabrication Methods for Protein and Peptide Features

A range of large-scale lithographic methods have been demonstrated for the generation of micro- and nanoscale protein and peptide biochips. In terms of photolithography, a great advantage of this method is its addressability (i.e., the ability to expose specific arbitrary locations), so different proteins can be site-selectively immobilized on different areas of the device after subsequent exposure-immobilization steps.^125,127^ Since proteins generally do not interact with light, several methods have also been described for the direct exposure of protein films while retaining protein structure and function.^112,131^ Photolithography further offers the advantage of being applicable to both thin and thick (e.g., hydrogels) film materials, provided the material is transparent to the appropriate wavelengths being employed. Even so, the wider application of these methods to other proteins should in all cases incorporate the appropriate validation experiments to confirm that the immobilized proteins retain their desired biological function.

Photolithography can of course be employed for the fabrication of substrates, which in turn can be exploited with other protein deposition methods. In the case of *in situ* translated NAPPA, the lithographic fabrication of “nanowells” to confine the produced proteins has enabled higher feature densities compared to arrays produced from planar substrates (giving up to a 4-fold increase in feature density^12,123^). In principle, however, other lithography methods could be employed to fabricate these nanowells and methods with a higher resolution, such as NIL that may in future yield even greater feature densities.

In comparison, μCP can be used for the direct deposition of proteins onto the substrate (i.e., additive fabrication), though both direct and indirect methods have been reported. Nanometer feature resolutions are also possible with these methods.^72^ Moreover, μCP has the great advantage of avoiding harsh chemicals and conditions, such as UV-light or high temperatures, during the fabrication process, with less difficulty in retaining protein activity compared to photolithography and NIL.

As mentioned in the section on lithography of oligonucleotide features, NIL is the large-area lithographic method of choice when high resolutions (<100 nm) and high feature densities are required.^111^ One limitation of NIL is that it is typically not used to directly print proteins, but to create the patterns that template subsequent protein immobilization. Nevertheless, direct molding has been recently obtained using protein film as a conformable material,^137^ even if this approach seems unlikely to be applicable to many proteins.

### Lithography of Cell-Based Features

Cell microarrays are devices that allow for the interrogation of living cells immobilized on the surface of a solid support. In these arrays, individual features may capture individual cells, or more commonly colonies of cells in 3D architectures.^138,139^ These devices can then be used for studies of cellular physiology, cytotoxicity, drug screening, and tissue engineering.^138,140^

### Indirect Lithography for Cell-Based Feature Fabrication

Due to the delicate nature of living cells, direct patterning remains a significant challenge even with soft lithographic methods. Thus, the typical approach whereby such arrays are fabricated is by printing the surface with various materials (including proteins or peptides using some of the methods described above) that can direct the attachment of cells on specific areas of the device.

**Figure 10:**
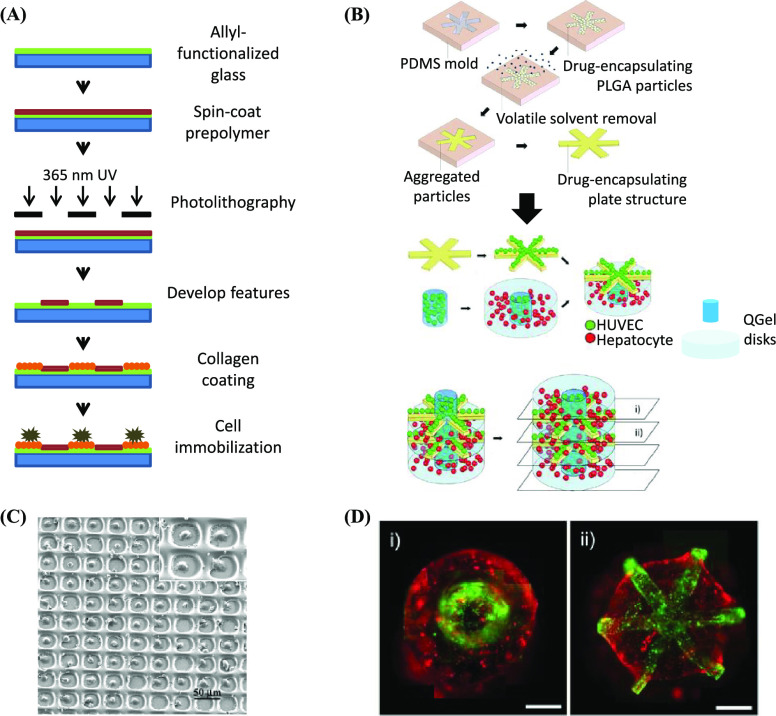
(A) Process for the fabrication of PEG hydrogel microstructures. Redrawn by the authors from ref (141). (B) Schematic representation of the process used to produce a liver lobule organoid. (C) Representative SEM image of the array of single fibroblasts with 91% cell occupancy (×150) generated with the process in (A). The inset shows a higher-magnification image of confined fibroblasts (×1200). Reproduced from ref (141). Copyright 2003 American Chemical Society. (D) Cross-sectional fluorescent images of the engineered liver lobule, where hepatocytes and HUVEC were indicated as red and green (scale bar: 300 μm). (B) and (D) adapted with permission from ref (142). Copyright 2012 John Wiley and Sons.

In one example, proximity photolithography was employed to initiate the polymerization of PEG-diacrylate on allyl-functionalized glass slides, to create grids of PEG-polyacrylate hydrogels that were resistant to protein and cell adhesion (i.e., negative tone, Figure 10A).^141^ The unexposed areas were then coated with collagen to promote cell adhesion. By using the polymer grids to confine 30 × 30 μm^2^ “wells”, it was demonstrated that arrays of a single fibroblast or hepatocyte cell per well could be produced. In this case, the size of these wells was chosen to match the size of the mammalian cells, and does not represent a limit of the microfabrication process.

However, 3D microstructures with embedded cells are of particular interest in the area of cell biology and tissue engineering, since hydrogels more closely mimic the microenvironment of the extracellular matrix *in vivo*.^143^ As a result, a number of lithographic methods have been researched to enable the fabrication of such structures.

In one example, μTM was used to pattern 3D features consisting of Matrigel, an animal-derived proprietary hydrogel that is widely used for cell culture.^144^ Here, PDMS stamps coated with poly(2-hydroxy-ethyl methacrylate) (poly-HEMA) were used to pattern a layer of Matrigel on a glass slide. It was found that the poly-HEMA coating was crucial as it reduced the adhesion of the Matrigel to the stamp upon lift-off and enabled the formation of high-aspect-ratio structures. It was reported that features with an 80 μm height and 100 μm width could be produced using this process. Even so, good pattern fidelity was difficult to achieve using this approach, with residual Matrigel often found in unwanted areas surrounding the patterns. These Matrigel structures were then seeded with epithelial cells, and it was shown that they organized into 3D epithelial tissue, demonstrating that the hydrogel provided an environment that mimicked the extracellular matrix and enabled tissue organization.

This self-organization of cells is particularly significant in the tissue engineering of “organoids”, where colonies of cells recapitulate the complex architecture of an organ. However, the production of such organoids requires the fabrication of biomaterials scaffolds composed of multiple components. For this purpose, REM has been used to produce millimeter-scale molded components with micrometer resolution.^142^ Here, REM (with a PDMS stamp) was used to prepare ∼1 mm star-shaped structures with a thickness of 150 μm consisting of poly(lactide-*co*-glycolide) (PLGA) impregnated with vascular endothelial growth factor (VEGF). Several of these structures were then sandwiched between a QGel disks (a proprietary PEG-based matrix metalloproteinase-sensitive hydrogel), within which human umbilical vein endothelial cells (HUVEC) and hepatocytes were able to organize into liver lobule organoids (Figure 10B). The use of REM in this application was particularly suitable, as it is capable of generating relatively large (millimeter scale) 3D objects at scales sufficient to construct the PLGA-QGel sandwiched structures.

### Direct Lithography of Cell-Based Materials

Despite the difficulties in attempting to apply lithography processes for the direct patterning of delicate living cells, examples of the lithography of materials containing live cells have recently been reported.

A photolithographic approach has been reported for the fabrication of a gelatin-methacrylate (GelMA) hydrogel layer with breast cancer cells embedded within the hydrogel to study cell migration.^145^ Here, a process involving two photolithographic steps was described, whereby the live cells were mixed with a GelMA prepolymer solution placed into a spacer with 500-μm-diameter features, 750 μm spacing, and a depth of 100 μm. This layer was then patterned by contact photolithography to polymerize the methacrylate groups, generating solid hydrogel discs within which were embedded the cells. Washing of the unpolymerized materials, application of a fresh GelMA (without cells), and a second round of photoexposure then furnished the completed cell array in a continuous hydrogel layer. A particular feature of this approach was that since two photolithographic steps are used, they can be separately tuned to give different degrees of cross-linking (and hence gel stiffness) between the two areas. Considering that matrix stiffness is a crucial biophysical aspect of the tumor microenvironment, this approach enables the study of cell behavior as they transition between matrices of differing stiffness. Using this approach, it was found that the cells had around 93% viability upon encapsulation, which decreased to ∼82% after 5 days of culture, indicating that the dose of UV light employed (360–480 nm, 800 mW cm^–2^) and the presence of prepolymer material had minimal effect on overall cell survival. Though this cell array was produced to study cancer cell migration, the same fabrication methods could be used to make embedded cell arrays for other applications such high-throughput drug screening and the development of personalized medicine.

**Figure 11:**
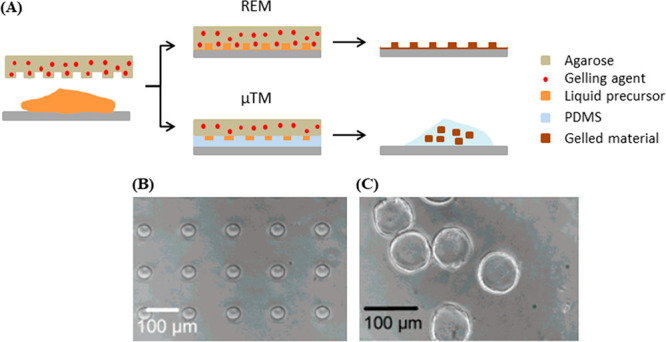
(A) Scheme of the two controlled-release molding processes, REM and μTM. Redrawn by the authors from ref (146). (B,C) SEM images of the (B) patterns produced with REM and (C) microparticles produced with μTM. (B) and (C) reproduced from ref (146). Copyright 2006 American Chemical Society.

REM and μTM have also been used for the fabrication of alginate or chitosan hydrogel microstructures containing live cells.^146^ To produce the alginate structures, the liquid pregel with cells was shaped using an agarose gel stamp impregnated with a gelling agent (CaCl_2_ solution), whereby the pregel was solidified in the mold by diffusion of the calcium out of the mold and into the alginate (Figure 11A). The same process was demonstrated for chitosan, which was gelled upon contact with agarose impregnated with a 5% w/v NaOH solution (high pH). A variation of this concept, where the stamp was used as a mold in a similar way to μTM, allowed the fabrication of microparticles upon release of the hydrogel from the mold (Figure 11B). Topographical features with lateral dimensions between 5 and 2000 μm and vertical dimensions between 10 and 200 μm could be fabricated using these methods. Thus, the use of chemical gelling here provides an orthogonal approach to photolithography, by allowing access to the fabrication of materials that are not photoreactive and avoiding exposure of the cells to UV light that may be damaging. Indeed, the entire fabrication process can be carried out under mild physiological conditions, maintaining high cell viability (>80%).

### Comparative Analysis of Fabrication Methods for Cell Patterning

In general, indirect methods where the structures are fabricated prior to the introduction of the cells are readily achievable with minimal modifications of existing lithography methods. Nevertheless, photolithography, REM, and μTM have been demonstrated for the fabrication of microstructures containing live cells. These methods exploit the properties of photo- and chemically cross-linkable hydrogels to form the cell-containing structures, which mimic the extracellular matrix and offer an *in vivo*-like environment that is conducive to cell viability.

Stamping or molding methods (REM and μTM) are of particular note for the direct fabrication of structures containing live cells, since they are easy to implement, do not use physically or chemically harsh conditions, and can be used to create 3D objects of the size that is appropriate for housing cells within the structures. These cell-containing objects can then be used as components for the construction of more multicomponent architectures that mimic tissue organization.

Even so, soft lithographic methods have yet to reliably demonstrate throughputs that rival that of photolithography, which remains the benchmark technology. Efforts have therefore been made to apply photolithography directly to cell-containing materials, and some evidence of cell viability postexposure has been shown. However, more detailed studies are required since UV light may damage the cells’ DNA (potentially resulting in alterations in cell genotype and phenotype) without necessarily reducing cell viability.^128^

### Lithography for Biosensor Device Fabrication

In general, sensors are devices that perform signal transduction, taking one type of stimulus (e.g., optical, mechanical, electrical, or chemical) and converting it into another. In the context of biological sensing, “biosensors” typically convert a signal of biological origin to an optical or electronic signal that can be quantified, reorded, and analyzed by an external circuit for the purposes of gaining information about that biological system. The aim of this section is to highlight the fabrication processes for biosensors and their interaction with the biological components involved.

### Lithography of Solid-State Nanopores

Solid-state nanopore sensors are a type of device that mimics biological ion channels and have been identified as a potential tool for the detection and analysis of individual biomolecules. In these sensors, biomolecules are electrophoretically driven through a nanoscale pore (0.5–100 nm), and the transit of the biomolecule is detected as a transient change in current.^147^ By analyzing the current signature (e.g., magnitude and timespan), physicochemical information on the molecule can be obtained (e.g., conformation, polarity) from which its structure can be inferred.^6,148^ The use of nanopore-based devices for DNA sequencing is now well-established, and active efforts are ongoing for a range of other applications.^147^

**Figure 12:**
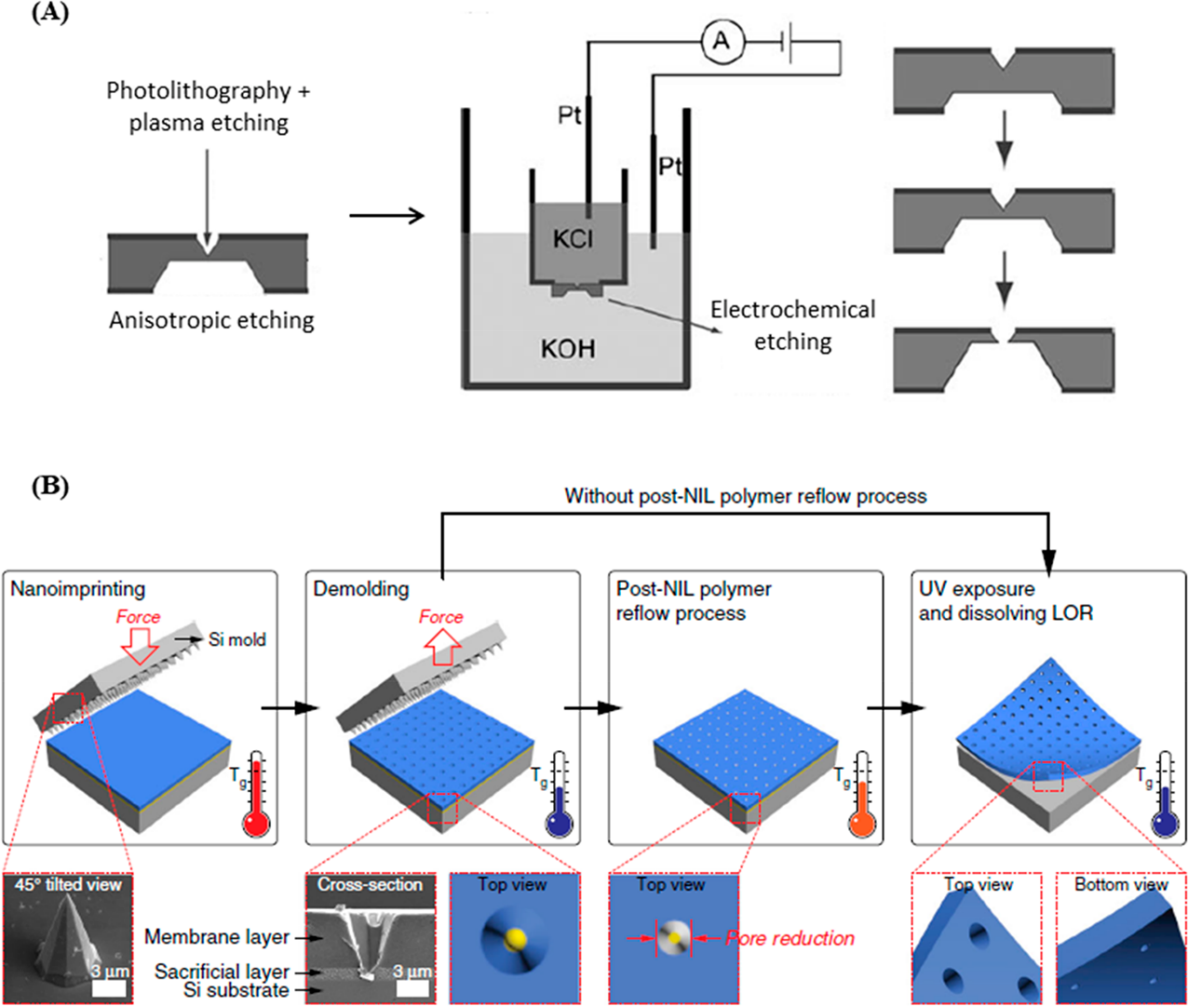
(A) Fabrication of silicon nanopores with photolithography and plasma etching, followed by anisotropic etching and electrochemical etching. Figure adapted with permission from ref (153). Copyright 2006 John Wiley and Sons. (B) Schematic diagram for the fabrication of perforated nanopores in a freestanding polymer membrane via NIL and polymer reflowing. Figure adapted with permission from ref (6). Copyright 2019 Springer Nature.

The general approach toward the fabrication of nanopore-based devices centers on the generation of pores of the desired size on the substrate, which is typically directed by various lithographic methods (and may include etching steps). The overall fabrication process must therefore be capable of achieving precise pore sizes with high aspect ratios. It should also ideally be capable of producing a range of pore sizes and shapes. Currently, EBL remains the most widely used method for addressing the location(s) of the nanopores on the substrate, since it can easily reach resolutions <10 nm. The initial pattern generated by EBL is then used to template a subsequent etching process, either by reactive ion etching (RIE) or by electrochemical etching, to “drill” through the substrate and generate the pore.^149−152^ In order to achieve large-scale device fabrication, efforts have turned toward harnessing photolithography. Indeed, examples of solid-state nanopore sensors produced on SiO_2_ and Si_3_N_4_ wafers with diameters of up to 10 cm have been reported.^153,154^ In order to generate these pores, photolithography followed by plasma etching is employed to generate inverted pyramids on one side of the wafer. Subsequently, anisotropic etching is used to obtain truncated pyramids on the opposite of the wafer. Finally, finely controlled electrochemical etching is used to open a nanopore between the two sides, with diameters down to 20 nm (Figure 12A). Devices made using this process were then verified to be suitable for the study of DNA translocation through the pore,^153^ and to investigate DNA length by discriminating the capture rate of the different DNA molecules in the nanopore, achieving a limit of detection down to 200 bp (the smallest DNA length difference tested).^154^

To date, there has also been one example of the application of thermal NIL to fabricate polymer-based nanopore sensors.^6^ Here, a silicon “microneedle” mold with a tip diameter of 25 nm and height of 9 μm were imprinted on a double resist layer via lift-off resist (LOR) and SU-8 resist above the LOR. The NIL is followed by a heating step to reflow the polymer, which resulted in smaller and more consistent pores. Subsequently, the SU-8 layer is cured with UV light and released by dissolving the LOR layer, generating a freestanding SU-8 membrane with nanopores (Figure 12B). Using this process, arrays of 6 and 12 nm nanopores could be produced. In contrast with other methods, this report is significant, as it demonstrates the fabrication of nanopores on a low-cost soft material.

### Lithography of Plasmonic Nanostructures

The field of plasmonics has advanced immensely over the years and has now transitioned from a topic of fundamental research to practical applications. In the area of sensing, alterations of a metal’s surface plasmons upon binding to an analyte can be quantified (by spectral changes to the incident light on the metal) and used to detect and quantify that analyte. Plasmonic-based sensing is particularly attractive in biosensing, since it does not require the analyte to be derivatized with an easy to detect moiety (i.e., it is “label-free”). In practical terms, avoiding the need for a labeling step means than any biomolecular analysis can be simplified, saving time, cost, and analyte; and avoids the risk that the labeling may alter the properties of the analyte.^155,156^ Furthermore, the fabrication of arrays of plasmonic devices can be readily carried out on a single substrate to enable multiplexed sensing (i.e., sensing of multiple analytes simultaneously).^31,157^ Two general approaches have been described: (1) sensors employing propagating plasmons on a planar metal substrate, and (2) sensors that employ nanoscale structures that have a localized surface plasmon resonance (LSPR).^158−160^

**Figure 13:**
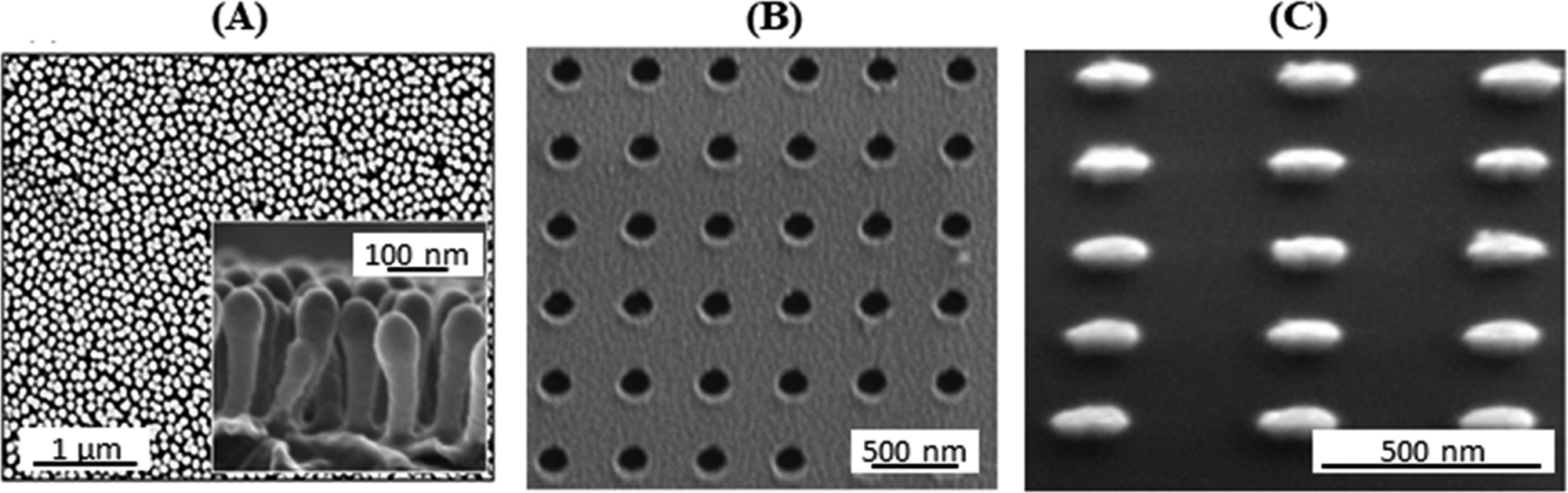
SEM images of nanostructures generated with NIL, including (A) nanopillars, (B) nanoholes, and (C) nanodiscs. (A) reproduced from ref (83). Copyright 2012 American Chemical Society. (B) adapted from ref (161). CC BY 4.0. (C) adapted with permission from ref (2). Copyright 2010 Elsevier.

Large-area lithographic methods, especially NIL, are particularly applicable to the fabrication of nanoplasmonic sensor devices, since they typically require large arrays of metallic nanostructures for LSPR sensing. In one example of a LSPR biosensor, thermal NIL is used to pattern an array of nanoscale high-aspect-ratio cylinders (“nanopillars”) consisting of cyclo-olefin polymer, which were subsequently coated with a gold layer to give plasmonically active structures (Figure 13A).^83^ Here, the thermoplastic polymer is used as part of the device structure, instead of being only exploited as a temporary resist material. Highly dense nanopillars with diameters between 30 and 70 nm and pitches of approximately 200 nm were obtained. The gold layer could then be further conjugated with anti-human-immunoglobulin G (IgG), which enabled the selective binding of IgG and hence its detection. It was subsequently found that the device achieves an IgG detection limit of 1.0 ng mL^–1^.

In another example, thermal NIL was used to fabricate a substrate with an array of nanoscale holes for LSPR-based protein sensing (Figure 13B). In this sensor, adsorption of the analyte to the array surface results in a shift in the wavelengths transmitted through the “nanohole” array.^161^ The fabrication of these arrays followed the standard NIL imprinting process (with a silicon stamp) of a thermoplastic resist coated on glass wafers (Figure 2G). After the imprinting and etching of the residual resist layer, a 50 nm gold layer is deposited followed by the resist lift-off, resulting in a grid of 185-nm-diameter nanoholes with a periodicity of 450 nm. Using this biodevice, the detection of BSA adsorption onto the nanohole array was demonstrated, with a sensitivity of 126 nm RIU^–1^. This value is similar to that which can be achieved by arrays manufactured with EBL^162,163^ and much higher than the values obtained with non-nanostructured sensors (∼500–12000 nm RIU^–1^).^164^ These results demonstrate that NIL is a viable alternative to EBL for this application, with implications for higher throughput and lower cost.

In order to further reduce the cost of the overall imprinting process, UV-NIL processes have been introduced as an alternative to conventional (thermal) NIL to produce plasmonic nanostructures. The cost advantage arises from its use of PDMS stamps that are cheaper than the rigid molds used by thermal NIL, and can be used several times at very low contact pressures (typically <1 kbar), while maintaining high resolutions and reproducibility.^2,165^ In one example, this method was used to generate gold nanodiscs (Figure 13C) for the plasmonic sensing of antibodies, where the imprinting was performed on a proprietary UV-curable AMONIL resist layer deposited on a PMMA resist.^2^ Using 365 nm UV exposure, nanoholes of 160 nm diameter and a periodicity of 500 nm were obtained in the AMONIL layer. After etching the residual AMONIL layer and the underlying PMMA, a gold layer was deposited. Finally, PMMA on unpatterned areas was removed, furnishing an ordered array of gold nanodiscs. These discs were then immersed in a solution containing thiolated polypeptides modified with a biotin molecule that can be recognized by anti-biotin antibodies. These devices were found to be extremely sensitive with the best results achieving limits of detection of 1.02 × 10^5^ antibodies, on 30 × 30 μm^2^ surface areas, which correspond to just 30 antibodies per nanodisc.

NIL can also be applied to fabricate more complex multilayered structures that exhibit enhanced LSPR, which in turn can be used to enhance the fluorescence emission of molecules bound on these structures. Thus, they can be used to increase sensitivity of conventional fluorescence-based assays. These structures generally consist of features ∼100 nm in width, assembled from alternating noble metal (e.g., gold) and dielectric films (e.g., SiO_2_). As an example, NIL has been used to fabricate an array of nanodiscs consisting of an Au and SiO_2_ sandwich structure (Au/SiO_2_/Au), to enable LSPR-enhanced fluorescent detection of proteins.^166^ Such nanostructures are obtained by the coating of glass surfaces with PMMA and a UV cross-linkable polymer imprinted with UV-NIL. Both the remaining UV-polymer and underlying PMMA are then etched, and alternating films of Au/SiO_2_/Au are deposited. The final PMMA lift-off furnished the array of multilayered nanodiscs, each of which was 100 nm in diameter with a periodicity of 500 nm.

In order to demonstrate enhanced protein detection, these discs were functionalized with anti-IL-2 antibodies. Here, the binding of IL-2 to allophycocyanin (APC) conjugated anti-IL-2 antibodies results in a strong fluorescence emission when imaged by optical microscopy. In the presence of nanodiscs, the fluorescence signal was enhanced 117-fold compared to areas lacking nanodiscs. Notably, this sensitivity was sufficient even to detect the quantities of IL-2 secreted by individual cells grown on these disc arrays, which enabled submicron resolution quantitative mapping of cytokine secretion.

### Lithography of Photonic Crystals

Photonic crystals are periodic dielectric nanostructures that are designed to either allow or block the propagation of electromagnetic waves of certain wavelengths, making them attractive optical materials for controlling and manipulating light.^15,167^ The propagation of light though photonic crystal structures results in a number of features that are potentially exploitable in biosensing. For example, a biochemical interaction (e.g., binding) on the photonic crystal surface causes a change in the effective refractive index that shifts the resonance wavelength peak, which can be correlated to target molecule concentrations with high sensitivity without time-consuming labeling procedures.^168^

**Figure 14:**
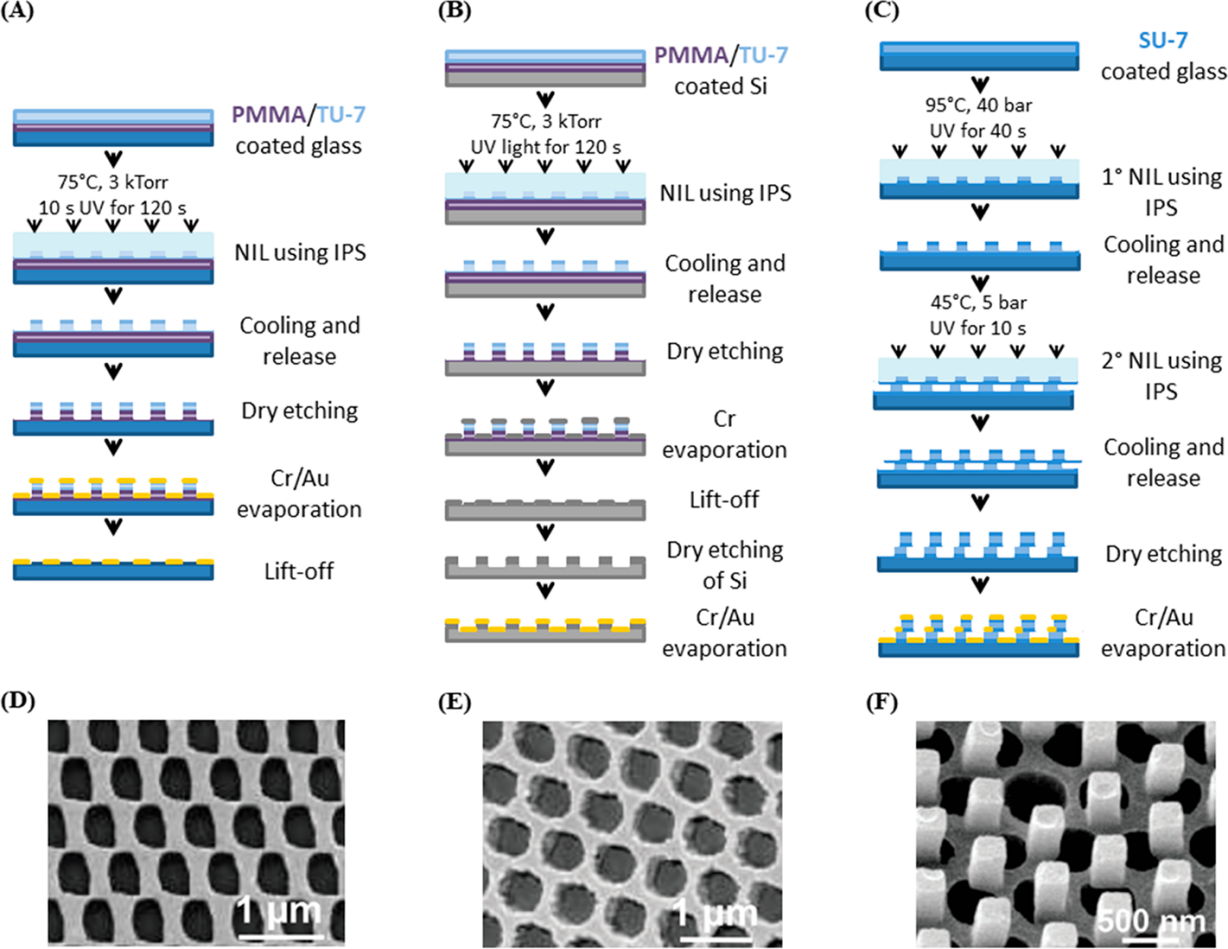
Schematic diagram of the fabrication process for (A) 2D, (B) quasi-3D, and (C) 3D photonic crystals by STU-NIL. Redrawn by the authors from ref (16). (D,E,F) SEM images of (D) 2D, (E) quasi-3D, and (F) 3D photonic crystals. (D,E,F) adapted from ref (16) with permission from The Royal Society of Chemistry. Copyright 2018.

Photonic crystal structures can incorporate different geometries, such as cavities, multilayered thin films, slabs, and pores, and different methods have been reported for their fabrication, including self-assembly and lithography.^168^ In this regard, the application of STU-NIL for the fabrication of a range of “2D” (i.e., monolayer materials with “zero” thickness), “quasi-3D” (1–5 periods thick), and thick “3D” plasmonic–photonic crystals nanostructures has been reported (Figure 14).^16^

To create “2D” arrays of nanoholes, NIL was use to imprint a TU-7 resist layer and that was coated on a layer of PMMA. After removal of the residual resist and PMMA by RIE, a thin film of Cr (2 nm) and Au (20 nm) was deposited. The removal of all remaining TU-7 then furnished the array of nanoholes on the film of Au/Cr (Figure 14A). Using this approach, nanoholes of various shapes with a size and pitch of 350 and 535 nm, respectively, were produced. To produce the quasi-3D structures, a thick layer of TU-7 was imprinted with the same nanohole shapes. RIE was then used to etch the resist and PMMA at different rates so that the holes were undercut (i.e., the bottoms were wider than the top). Cr was then deposited to ensure metal coating throughout the bottom of the nanoholes and used as a mask to subsequently etch the Si substrate. Thus, Cr/Au were evaporated on these quasi-3D nanoholes with 350 nm width and 535 nm pitch and 350 nm depth to generate two different plasmonic layers (Figure 14B). 3D nanostructures were obtained performing UV-NIL on an SU-8 resist that was placed on top of an existing quasi-3D nanohole array. RIE of the residual SU-8 followed by metal deposition then gave the final structures (Figure 14C).

These complex nanostructures confine and enhance electromagnetic field intensity through the hybrid coupling of plasmonic and photonic crystal modes, resulting in very large plasmonic enhancements of 276, 946, and 1376 nm RIU^–1^ for the 2D, quasi-3D, and 3D structures, respectively. In order to demonstrate biosensing, the 3D structures were functionalized with anti-epithelial cell adhesion molecule (anti-EpCAM) antibodies, which enabled them to bind vesicles (exosomes) derived from fibroblast cells. It was found that the detection of as low as 10^4^ exosome particles mL^–1^ of analyte sample was achieved. The ability to detect and quantify exosomes is significant, since they are released in higher quantity by cancer cells, thus representing a promising target for early cancer detection and diagnosis.

### Lithography of Components of Electrochemical Biosensors

Electrochemical biosensors transduce biochemical information, such as analyte concentrations, into an electrical signal (either a current or change in voltage) that can be recorded and analyzed.^169^ Large-scale lithographic techniques have been widely used to fabricate electrochemical biodevice components, such as channels or electrodes. In these cases, lithographic methods with high precision and resolution are necessary in order to construct devices that are highly miniaturized yet contain multiple components. For example, the i-STAT hand-held blood analyzer commercialized by Abbott consists of an array of microelectrodes that use photolithography during their fabrication process.^170^ The engineering of electrode surfaces is also important, since nanostructured electrodes can give rise to higher surface areas, which in turn permits higher performance with smaller electrodes. Furthermore, device miniaturization is also desirable for implantable devices where size is an important consideration.^171^

One application where high-resolution lithography is needed is in the fabrication of redox cycling sensors.^172−174^ These sensors detect the presence of redox active molecules by repeatedly oxidizing and reducing them between two closely positioned electrodes. This allows multiple reactions of a single molecule at the electrodes, resulting in an amplified electrochemical signal. The sensitivity of this method depends on the average number of cycles a molecule performs before it escapes from the volume between the two electrodes. Thus, it is strongly influenced by the geometry and size of the device, with typical distances between the two contacts of ∼65–230 nm. In the context of biosensing, these devices can be used for the direct sensing of redox active molecules such as neurotransmitters,^172^ or nonredox biomolecules that trigger an enzymatic generation of redox molecules.^173,174^

**Figure 15:**
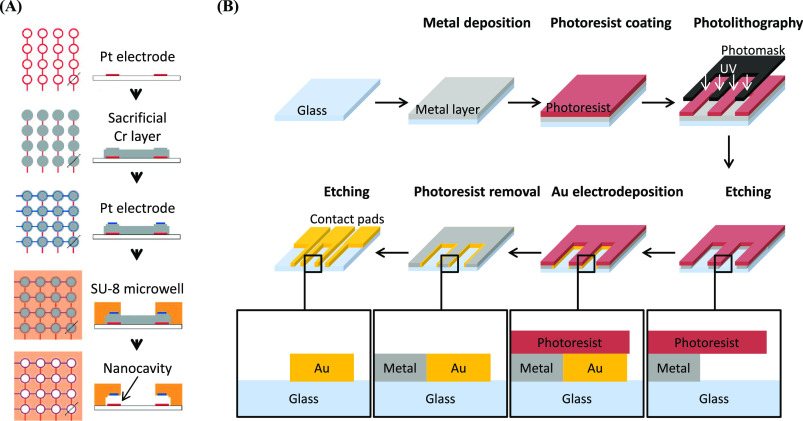
(A) Schematic diagram of the redox cycling sensor fabrication process. The photolithographic fabrication process leads to the generation of an array of individual electrochemical cells. Figure adapted with permission from ref (173). Copyright 2015 The Royal Society of Chemistry. (B) Process flow for the generation of gold nanowires by LPNE. Redrawn by the authors from ref (176).

In terms of the fabrication, photolithography is currently the method of choice since the multistep (and multiexposure) processes used to fabricate semiconductor devices can be adapted to generate the complex designs with internal cavities needed to separate the electrodes. In one representative example (Figure 15A),^173,174^ the fabrication first involved the deposition and photolithography of Ti/Pt bottom electrodes on a glass substrate, followed by a sacrificial Cr layer, and then Cr/Pt top electrodes. A SU-8 resist layer is subsequently spin-coated on the surface, and photolithography is used to pattern “microwells” with 150 μm diameter. Finally, the Cr sacrificial layer is etched to form nanocavities. By using nanocavities with a 190 nm distance between the electrodes fabricated by this process, limits of detections for endotoxins of 0.2 and 0.5 EU L^–1^ are obtained for reaction times of 1 h and 30 min, respectively.^174^ These values are 5- and 2-fold lower than those obtained with conventional endotoxin assays, making this biosensor highly sensitive.

Apart from the direct fabrication of device components, photolithography can be used to produce patterns in resist materials that can then act as a template for the electrodeposition of metallic features. This indirect approach enables the fabrication of objects with sizes or structures that are not accessible to photolithography alone. For example, by using a technique termed lithographically patterned nanowire electrodeposition (LPNE), metallic nanowires can be produced with dimensions smaller than the resolution limit of photolithography.^175,176^ In LPNE, the substrate is first coated with a sacrificial metal followed by a photoresist. Photolithography is then performed whereby the edges of the features define the final paths of the nanowires, and the sacrificial metal is then etched (Figure 15B). Crucially, the etching is performed such that the sacrificial metal is undercut to produce a “horizontal trench”, within which the desired metal can then be electrodeposited. Removal of the remaining resist and sacrificial metal then furnishes the final nanowires. In the best examples, nanowires with thicknesses of <10 nm over millimeter lengths can be produced.^175^

Another example of combining lithography with electrodeposition is the fabrication of nanostructured microscale electrodes.^177,178^ Starting with Au electrical contacts coated with insulating SiO_2_, photolithography was used to produce 500 nm apertures in the SiO_2_ to expose the underlying Au. Electrodeposition was then used to grow highly textured or dendritic Pd structures from these apertures. The affinity of Pd with thiols was then exploited to attach biomolecules, thus enabling the Pd to act as an amperometric electrochemical sensing element. It was demonstrated that the degree of nanostructuring changed the response to a given nucleic acid, with the most finely textured Pd (dentritic structures 20–50 nm) having limits of detection for the target as low as 10 aM.^177^ Photolithography was particularly advantageous in this case because multiple sensor units can be produced in parallel on the same substrate, for multiplexed sensing.

Recently, approaches for the ultrasensitive direct electrochemical detection of biomolecules (i.e., without the need of amplification) have been developed using advanced carbon-based nanomaterials such as single-layer graphene sheets. Here, the rate charge transfer (i.e., current) from the bulk electrolyte to a redox mediator (e.g., ferrocene) immobilized to the graphene can be measured by cyclic voltammetry, and is sensitive to alterations to the local environment. In order to harness this effect for biosensing, the coimmobilization of a biomolecular recognition element (e.g., DNA, antibody) that specifically binds the analyte of interest is also carried out, so that in the presence of the analyte, binding results in a change in current. For these sensors, improvements in sensitivity can be achieved by introducing artificial defects such as controlled edges to enhance graphene’s electrochemical properties.

In one example, UV-NIL was exploited for the generation of large areas of single-layer graphene “nanomesh” (i.e., graphene layer into which an array of nanoscale holes are generated)^179^ where dangling bonds were present on the hole edges. The high density of edges achieved through NIL thus results in bandgap separation and semiconductive properties that are necessary for sensing.^180^ To produce this nanomesh, a trilayer of AMONIL/Ge/PMMA on graphene was deposited and imprinted using a PDMS stamp, and the 260-nm-wide nanoholes were transferred on graphene through RIE. In order to demonstrate the detection of DNA, the graphene surface was derivatized with a polymer containing ferrocene, followed by the conjugation of single-stranded DNA. The presence of another single-stranded DNA that is complementary to the immobilized strand results in hybridization and a sensor response. The electrochemical response of the patterned graphene shows an enhancement in current density compared to nonpatterned graphene layer, giving limits of detection down to attomolar levels (femtomolar level with the unpatterned material).

As an example of μCP use in the fabrication of bioelectrodes, choline oxidase (ChOx) and GOx are directly printed onto individual electrodes (40 × 150 μm^2^ in size) of a microelectrode array to provide the first example of an enzymatic sensor of neurotransmitters.^5^ The proteins were deposited on a layer of polyphenylenediamine on the electrodes, exploiting electrostatic attraction to transfer and immobilize the proteins. Significantly, this work demonstrated that multiple proteins can be printed onto selected electrodes on the array by using two different stamps (one for each enzyme to be deposited), with each stamp conforming to the location of the electrodes where the protein would be deposited.

### Summary of Biosensor Component Fabrication

Large-scale lithographic methods are widely used in the fabrication process of various components of biosensor devices, e.g., electrodes, transduces, filters. The examples discussed show how these methods are able to produce materials with improved performance through the introduction of nanoscale features (e.g., nanoholes) compared to the unpatterned material, resulting in sensors with improved sensitivity, lower analysis time, and the amount of sample needed. Indeed, the fabrication of complex 3D photonic crystal designs by NIL represents a powerful example of how lithography methods can be employed to fabricate complex arbitrary designs.

However, many of the examples in the literature generally lack comprehensive testing of the biosensing that is necessary for practical applications. A full suite of control experiments is typically not shown. For example, in the graphene nanomesh DNA sensor noted above, control experiments using noncomplementary DNA sequences is not shown. Therefore, it is unclear if good sequence specificity can also be achieved, or whether the signal is simply the result of nonspecific adsorption of any DNA molecules to the graphene.

## Conclusions and Future Perspectives

The increasing interaction between device fabrication, chemistry, and biotechnology communities is leading to an increasing number of processes that can be used in large-scale biochip and biosensor fabrication, which will be necessary for the practical deployment of such devices in “real world” applications.

This review demonstrates that a variety of large-scale lithographic approaches have been applied for both direct and indirect fabrication of features containing biomolecules. In this regard, stamp transfer methods such as μCP are notable, as they can perform direct (i.e., additive) deposition of biomolecules and employ milder and more physiologically compatible conditions, compared to photolithography and NIL, which often include the use of UV-light exposure, high temperatures, and toxic solvents. There are also a few examples where the patterning occurred directly on DNA-protein or cell-based materials have been described, highlighting how “conventional” cleanroom methods can be adapted to pattern biomolecules. Examples of both positive and negative tone lithography of biomolecules have also been demonstrated. This capability is important, as it enables the flexibility for a wider variety of substrate designs.

Among large-scale lithographic methods, conventional photolithography is still the most mature and widespread lithographic technique when nanometer-scale resolution is not required. It can draw upon the well-established processing methods and offers very large area fabrication (Figure 1). In comparison, soft lithography and especially soft UV-NIL offer higher resolutions at lower cost. Indeed, comparable nanometer resolutions can be achieved through projection photolithography only with sophisticated and more expensive lens systems. On the other hand, projection photolithography offers the benefit of avoiding direct contact between the mask and the substrate, decreasing contamination and enabling the lithography of very soft materials.

There are still challenges that need to be addressed to increase the use of large-scale lithographic methods for manufacturing *ex vivo* biochips and biosensors. One of the main drawbacks of large-scale lithographic techniques is the use of EBL for fabrication of masters, which represents a major contributor to the time and cost of the entire process. Moreover, to develop more sustainable lithographic methods, the development of more bio-based materials is needed. A further complication in the fabrication of biochips containing biomolecules is that proteins and DNA exert their function only at specific interfaces where they bind with other biomolecules. Thus, the orientation of the binding site with respect to the substrate must be controlled for the realization of biochips with high performances.^181,182^

Currently, many papers where large-area lithographic methods are applied to device generation are still at the “proof-of-concept” stage, and not yet demonstrated at true manufacturing scale. Making chips or devices for real-world applications requires complex integration of many components, each of which may be fabricated by different methods (e.g., the tissue chips, biosensors). It must be acknowledged that a method for rapid mass production of any chip or device, at low cost with nanometer-scale resolution and precision, has yet to be realized. The examples discussed herein, which only employ one lithographic method, address only some of these idealized characteristics in the final biochip or biosensor (e.g., in resolution, throughput, compatibility with the biomolecule of interest). In the future, this might be overcome by further improvements in lithographic approaches and a better synergy between lithography, printing technologies (e.g., inkjet and screen printing, as well as 3D printing),^37^ and molecular self-assembly.^34,183^ Additionally, emerging methods such as multiplexed scanning probe lithography may in future be viable at the manufacturing scale.^184−186^ Indeed, this type of lithography has already been demonstrated at the 3 in. wafer scale, and could in future offer a route to low-cost “desktop fabrication” that is compatible with soft materials and biomolecules.

## Vocabulary

Lithography: the process of producing patterns on a surface
resist: a material that is used to transfer a pattern to a surface by preventing (“resisting”) the etching of the material covered by the resist material
self-assembled monolayer: a spontaneous molecular assembly on a surface by adsorption in an ordered way
cross-linking: the process of connecting two adjacent chains of atoms in a large molecule, such as a polymer, through a chemical bond
biomarker: a substance that is measured as an indicator of normal biological processes, pathogenic processes, or pharmacological responses to therapeutic intervention
scalable: the ability of a system to maintain or increase its level of performance or efficiency even as it is tested by larger operational demand
